# Incidence of HIV in Sub-Saharan Africa, 2000–2015: The Interplay Between Social Determinants and Behavioral Risk Factors

**DOI:** 10.1007/s10461-021-03279-9

**Published:** 2021-06-05

**Authors:** Deepa Jahagirdar, Magdalene Walters, Avina Vongpradith, Xiaochen Dai, Amanda Novotney, Hmwe H. Kyu, Haidong Wang

**Affiliations:** Institute for Health Metrics and Evaluation, Seattle, WA USA

**Keywords:** Social determinants, Economic determinants, Epidemiology, Sub-Saharan Africa, HIV incidence

## Abstract

HIV incidence in sub-Saharan Africa declined substantially between 2000 and 2015. In this analysis, we consider the relative associations of nine structural and individual determinants with this decline. A linear mixed effects model of logged HIV incidence rates versus determinants was used. The data were from mathematical modelling as part of the 2019 Global Burden of Disease Study in 43 sub-Saharan African countries. We used forwards selection to determine a single final model of HIV incidence rate. The association of economic variables and HIV knowledge with incidence was found to be driven by education, while ART coverage had the largest impact on other determinants’ coefficients. In the final model, education years per capita contributed the most to explaining variation in HIV incidence rates; a 1-year increase in mean education years was associated with a 0.39 (− 0.56; − 0.2, t = − 4.48 p < 0.01) % decline in incidence rate while a unit increase in ART coverage was associated with a 0.81 (− 1.34; − 0.28, t = − 3.01, p < 0.01) % decline in incidence rate.

**Supplementary Information:**

The online version contains supplementary material available at 10.1007/s10461-021-03279-9.

## Background

The global HIV epidemic underwent a series of transformations since the turn of the century. The introduction and wide disbursement of antiretroviral therapy (ART) drove a decrease in deaths and incident cases of HIV in many Sub-Saharan African (SSA) countries. In 2000 ART coverage averaged 0% across SSA; by 2015, coverage averaged 43% across all SSA locations. The rise in ART was accompanied by a large decrease in the number of deaths in the regions, with a 27.85% reduction between 2000 and 2015 [1] .

Beyond ART, developments related to sexual behaviours, social determinants and awareness also assisted in influencing the HIV epidemic. Increases in condom usage, awareness of, and attitude towards HIV and HIV prevention are associated with declines in HIV incidence [2]. For example, in Kenya, information campaigns were found to be effective to increase the knowledge required to prevent HIV infection. This change was seen to reduce the risk of HIV infection [3]. Other determinants, such as school attendance, years of education completed [4] and lagged distributed income (LDI), a weighted average of Gross Domestic Product per capita [5], also impact HIV incidence. These factors have improved substantially in Sub-Saharan Africa since 2000 and can additionally have direct impacts on transmission and access to treatment [6]. For example, increased education and school attendance has been found to be associated with protective sexual behavior in women and girls, including an older age of sexual debut, lower numbers of sexual partners and use of condoms [4]. Though wealth is inconsistently associated with lesser HIV burden at an individual level [7], improvement in traditional economic indicators are associated with reduced HIV [5] and are also considered indicators of overall health system quality [8]. Conversely, high levels of sexual violence have been found to often drives higher HIV rates in women [9].

While HIV-related behavioral change and social determinants of HIV have often been explored, less work has characterized the interplay between these variables. Most often, studies focus on the impact of one risk factor and/or several variables at once on HIV outcomes. However, this approach precludes understanding the relationships between factors [10, 11] which can also drive their association with HIV incidence. Quantifying variables that are operating through other factors is relevant to planning as well as reducing the risk of biased estimates in causal analysis of risk factors on HIV outcomes [12]. For example, the change in the impact of education on incidence when we adjust for HIV knowledge scores could enable understanding factors’ explanatory power, as well as contribute to further studies that rely on understanding mechanisms.

This analysis examines the associations between various sociobehavioural and economic factors on HIV incidence rate, when sequentially controlling for each other. We investigate the nuances of the relationships between these factors and HIV incidence and select the most parsimonious model of HIV incidence. Specifically, we sought to: (i) Describe the relationships between socio-behavioural factors and economic determinants known to influence HIV incidence; (ii) Quantify the association between these factors and HIV incidence rate.

## Methods

### Sample

We included 43 countries in our analysis with yearly data on HIV incidence and covariates from 2000 to 2015. These countries comprise the Sub-Saharan Africa super region as defined by the Global Burden of Disease studies, which have experienced a disproportionate HIV burden in the past three decades.

### Covariates

We included economic/structural and socio-behavioral factors, as described below.

Economic and structural factors included lagged distribution income (LDI), HIV cure and treatment financing per capita (henceforth HIV spending) and education per capita. LDI is the weighted average of GDP per capita for a 10-year period preceding the year of interest. Decreasing weights are given to GDP per capita values from years further away from the year of interest. The estimates of spending on HIV care and treatment used herein (unpublished) are similar to those published previously [13, 14].^1^ Education was represented in this study by average years of education received in adult age groups. We also included the prevalence of non-partner sexual violence. Both were estimated as part of the Global Burden of Disease Study [15].

HIV-specific knowledge and attitude scores and modern contraceptive use were additional covariates. Briefly, HIV-specific knowledge was based on individual-level data for 12 HIV knowledge and attitude indicators (yes/no questions) including, for example, knowing where to obtain an HIV test, that condom use prevents HIV, and that having one partner reduces HIV risk. The number of ‘yes’ answers to these questions formed the bases of the knowledge and attitude scores. This information was extracted from 267 surveys and collapsed to country-level estimates (Online Appendix Table 1). A spatiotemporal Gaussian progress regression was then used to provide a complete time series since year 2000 for knowledge and attitude scores at the country-level for all countries in sub-Saharan Africa [16]. This model allowed us to borrow strength from ‘data-rich’ time and space points to fill in the sparser locations and years.

Modern contraceptive prevalence was the proportion of all women of reproductive age (15–49 years) who are currently using, or whose sexual partner is currently using, at least one form of modern contraception. Methods included male or female sterilization, male or female condoms, diaphragms, cervical caps, sponges, spermicidal agents, oral hormonal pills, patches, rings, implants, injections, intrauterine devices (IUDs), or emergency contraceptives [17]. Detailed description of the estimations of these covariates can be found elsewhere  [18, 19].

Finally, ART coverage rate was measured as the total number of people on ART divided by total prevalence in each country-year. ART and prevalence were an additional output of the model for incidence rates described under *Outcome*.

### Outcome

The HIV incidence rate per person among all ages and both sexes was estimated for the 2019 Global Burden of Disease (GBD) Study using the Estimation and Projection Package-Age Sex Model (EPP-ASM) [20]. This model was originally developed by the UNAIDS Reference Group on Estimates, Modelling, and Projection, and extensively built upon as part of the GBD [1], specifically to model HIV outcomes. EPP-ASM determines incidence at every time point as a function of the transmission rate among the un-treated population. It uses available data on HIV, prevalence, ART coverage and population demographics.

Transmission rate was modelled using the ‘r-hybrid’ model, in which transmission follows a logistic function until 2003, a linear interpolation until 2008, and a random walk process thereafter. These different forms reflect the initial spike and peak of the HIV epidemic, followed by a period of stabilization. Demographic and treatment inputs including population, migration, fertility rates, and treatment progression rates inform the integrated natural history model, allowing for a complete population projection of HIV incidence, prevalence, deaths and ART coverage in every year, age and sex.

The demographics inputs were from GBD 2019 Demographics estimates, while treatment inputs were from UNAIDS public release country files. Prevalence data were from country-reported antenatal care (ANC) clinics and nationally-representative household surveys. These consist of both Demographic and Health Surveys with HIV testing and Population-based HIV Impact Assessment (PHIA) surveys (data sources by country are available in Online Appendix Table II). The number of people on ART and prevention of maternal to child transmission are also reported to UNAIDS by countries in publicly available data. These are used as in inputs in this model. The methods have been previously described in full detail  [21].

### Analytic Method

To describe trends in incidence, we derived the percent change in the HIV incidence rate between 2000 and 2015 for each country in our sample. The evolution of variation across countries was explored using the standard deviation of incidence rates.

We then developed a model of incidence rate as a function of the independent variables described under *Covariates*. First, crude correlations between predictor variables in 2000 were assessed. We employed a rule of thumb of 0.8, meaning when a pair of variables showed Spearman rank correlations of 0.8 or higher, we excluded one of these from further analysis. We then modelled the association between the covariates and the outcome using a linear mixed effects model that accounted for within country correlation in incidence across years for all analyses as in Eq. (1).

1 $$Y_{it} = \beta K_{it} + \left( {\lambda + u_{oit} } \right)X_{it} + \varepsilon_{it} + u_{1i}$$

where Y is the log incidence rate per person in country *i* at *time* t, K is a vector of country and time specific covariates with estimated coefficients $\beta$, *u*_1*i*_ is the country-specific random intercept term, and ε_it_ is the random error. A priori, we found that trends in LDI, *X*_*it*_, varied across countries, so we allowed a country-specific deviation, *u*_*oit*_, from the coefficient λ on this variable in all models, i.e. a random slope.

In a modified forwards selection procedure [22], we sequentially added each covariate, starting with an intercept-only model. Traditionally, in an automated procedure, previously added covariates are dropped when adding another one means it no longer helps to improve the model, i.e. all covariates in the model do not meet some criteria. However, prior to dropping covariates, we determined the impact on all the coefficients when each covariate was added. This allowed describing which variables were acting as confounders. We then dropped covariates step-wise that were observed to lose statistical significance after adding another covariate. Rather than relying entirely on significance which is problematic due to underestimation of standard errors in selection procedures [23], we employed other model comparison metrics including the Akaike Information Criterion (AIC), the Bayesian Information Criterion (BIC) and model deviance to confirm they remained stable after covariates were dropped.

We determined the relative magnitude of the covariates’ contributions to explaining variation in the outcome using each variable’s sums of squares from an ANOVA decomposition of fixed effects [24]. The sum of squares for each fixed effect in the model, and an F statistic (the sum of squares divided by the residual variance times the degrees of freedom) was used to help to informally judge significance.

Uncertainty from the EPP-ASM HIV model described under *Outcome* was propagated into the mixed effects model. We ran the mixed effects models 1000 times each, each time drawing one of 1000 resampled outputs of the HIV statistical model. The final standard error was derived as:

$$SE_{final} = E(\varepsilon_{it} + u_{i} ) + SE\left( {\emptyset_{x} } \right)$$

where $\emptyset_{x}$ is coefficients estimated on each × draw (1–1000). The result was an inflated final variance that accounted for both uncertainty in the outcome from the HIV model and uncertainty from the mixed effects model.

All analyses were performed in R [25] using the *lme4* package [26].

## Results

### Country Characteristics

**Fig. 1:**
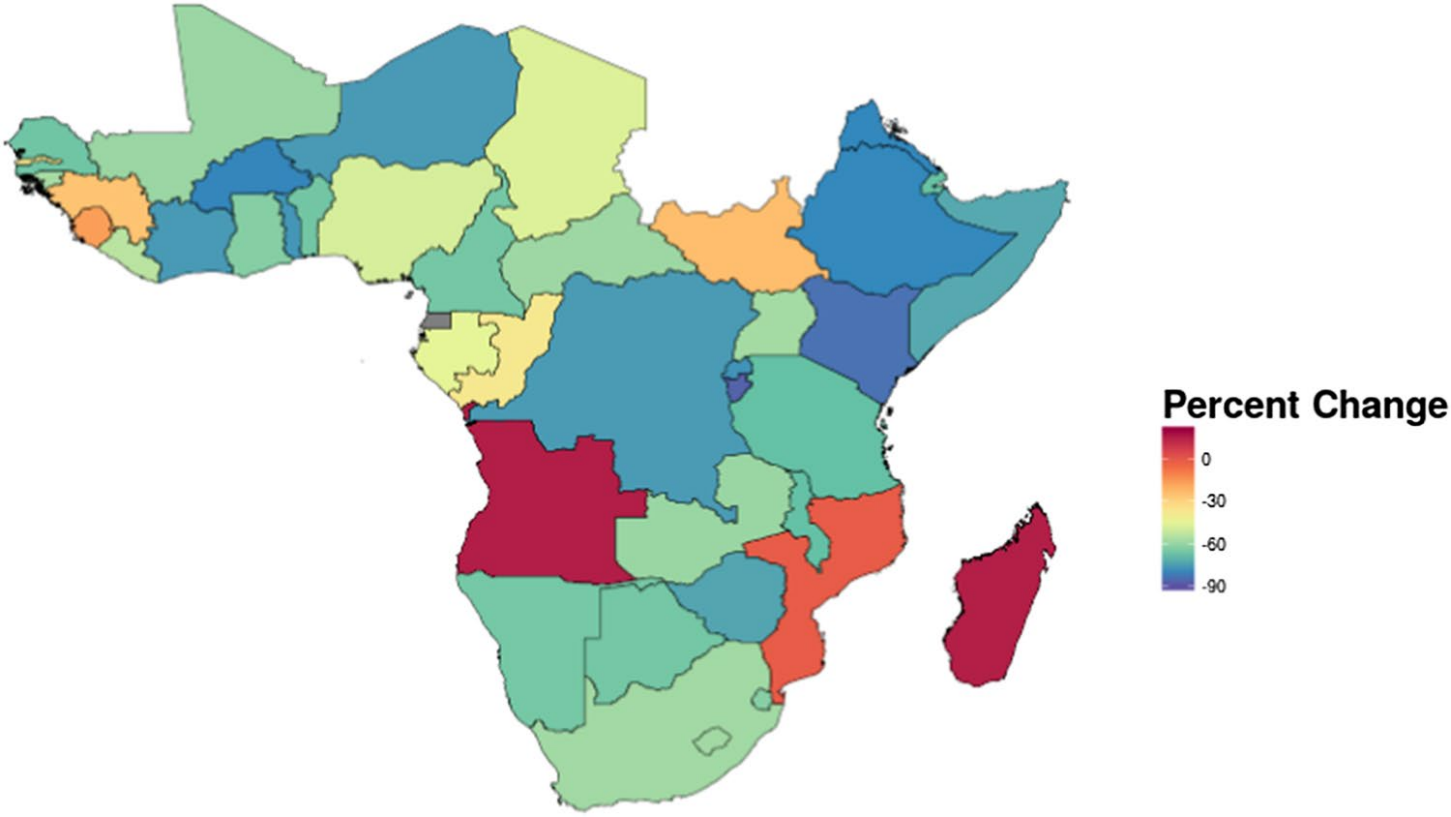
Percent change in mean incidence rate in 2000 versus 2015

Overall, the mean HIV incidence rate declined from 6.15 per 1000 (SD = 0.54) in 2000 to a mean of 2.35 per 1000 (SD = 0.17) in 2015. All countries except three (Angola, Madagascar, Equatorial Guinea) experienced a decline over this time period, though the percent changes ranged from − 86 to 51 percent (Fig. 1) The decline in standard deviation demonstrates the gains were not skewed. Characteristics and percent changes by country are available in Online Appendix Table III. Incidence time series are visualized in Online Appendix Figure I.

The theme of overall improvement during 2000–2015 was also evident in the trends of most of the covariates. All covariates improved except non partner sexual violence which increased slightly between 2000 and 2015. ART rates were effectively zero on average in 2000 but rose to 38% by 2015, while LDI increased by 62.9%, spending on HIV by 194% and contraception prevalence by 85%, from 12% in 2000 to 23% in 2015 (Table 1).

**Table 1:** Mean percent change between 2000 and 2015 for 43 countries in sub-Saharan Africa

Covariate	Mean 2000 (SD)	Mean 2015 (SD)	Percent change
Incidence rate	6.15 (10.05)	2.35 (2.99)	− 61.76
ART coverage	0 (0)	0.38 (0.17)	N/A
Economic determinants			
Lag-distributed income	2499.72 (2857.74)	4071.95 (6305.77)	62.9
Years of education completed	4.65 (2.01)	6.3 (2.14)	35.53
HIV curative care spending	1.88 (6.64)	5.52 (11.37)	193.5
Socio-behavioral			
Attitude score	0.53 (0.1)	0.65 (0.11)	22.02
Contraception prevalence	0.12 (0.12)	0.23 (0.15)	84.94
Knowledge score	0.55 (0.13)	0.75 (0.1)	36.89
Non-partner sexual violence	0.1 (0.07)	0.1 (0.07)	2.85

### Correlation Between Covariates

**Fig. 2:**
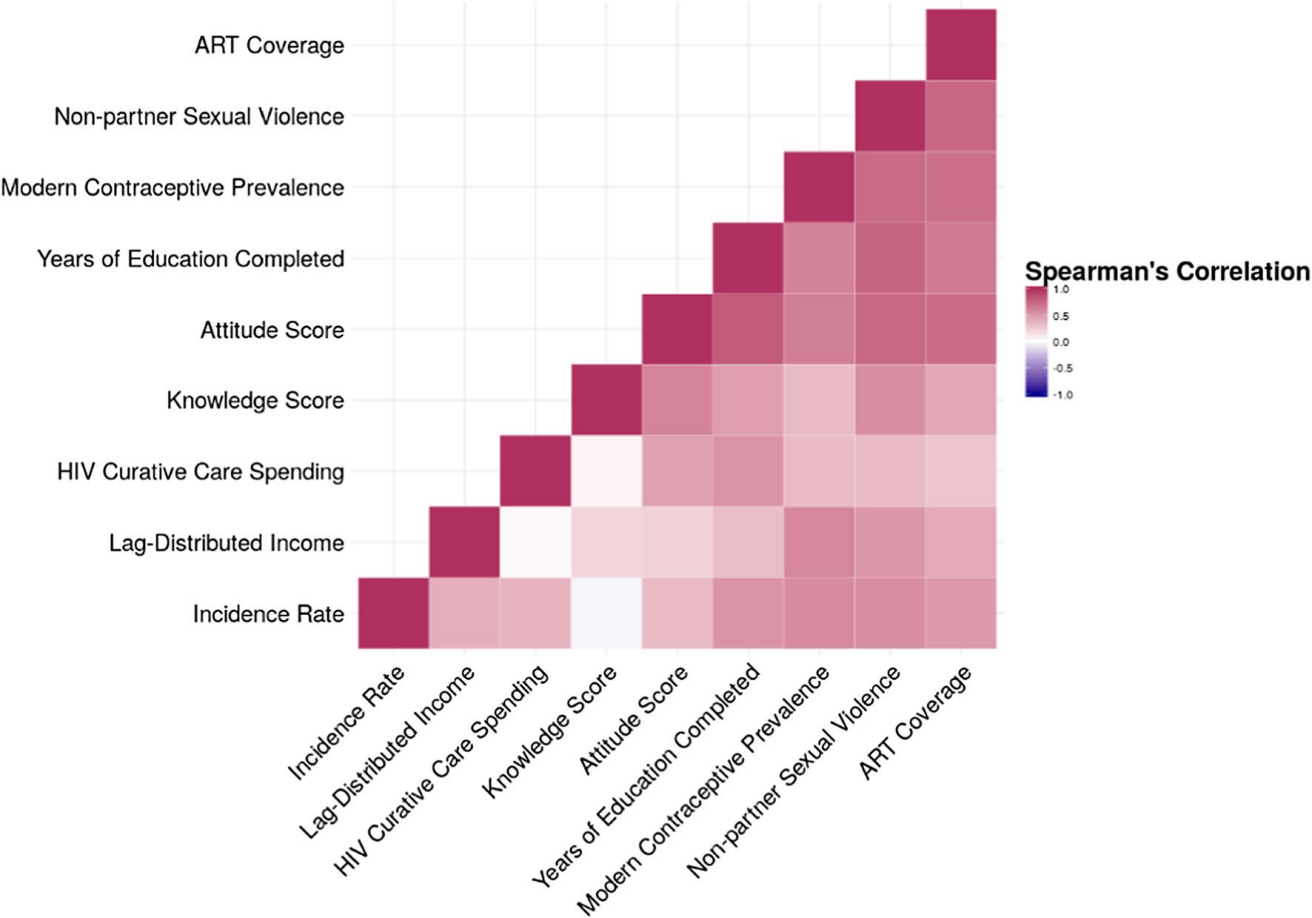
Spearman correlations between all covariates included in the analysis in the year 2000

The covariates had moderate to strong correlations with each other and independently with HIV incidence rate (Fig. 2). HIV-related knowledge and attitude scores were the most strongly correlated with each other (R = 0.82), thus we removed the attitude scores for the remaining analysis. All the other correlations were below 0.8, the highest being between HIV-related treatment spending and attitude scores with R = 0.77. There was virtually no correlation between ART coverage rates and non-partner sexual violence (R = 0.01), which was the lowest crude correlation observed. The crude correlations countered background knowledge; higher HIV incidence was associated with higher values of all covariates except for ART rates (Fig. 2).

### Results When Sequentially Adding Covariates

All results are displayed in Table 2. Sums of squares tables for each model are available in the Online Appendix Table IV, and model fit statistics for all models are available in Online Appendix Table V.

**Table 2:** Full sequential modelling results of the association between covariates and logged HIV incidence rate^a^

Model^c^	Intercept	Log lag-distributed income	Log HIV spending	Knowledge score	Years of education completed	Modern contraception prevalence	Non-partner sexual violence	ART coverage
Intercept only	− 5.82^*^ (95% CI: − 7.17;-4.48) t = − 8.48 (p = < 0.01)							
Log lag-distributed income	6.42 (− 2.91;15.74) t = 1.35 (0.16)	− 1.7^*^(− 2.97; − 0.43) t = − 2.63 (0.01)						
Log HIV spending	3.49 (− 4.91;11.88) t = 0.81 (0.29)	− 1.33^*^ (− 2.51; − 0.16) t = − 2.23 (0.033)	− 0.16^*^ (− 0.22; − 0.09) t = − 4.87 (< 0.01)					
Knowledge score	1.34 (− 5.68;8.36) t = 0.37 (0.37)	− 0.8 (− 1.75;0.15) t = − 1.66 (0.101)	− 0.05 (− 0.11;0.01) t = − 1.76 (0.09)	− 2.42^*^ (− 3.31; − 1.52) t = − 5.3 (< 0.01)				
Years of education completed	− 1.01 (− 7.18;5.16) t = − 0.32 (0.38)	− 0.38 (− 1.25;0.49) t = − 0.85 (0.28)	− 0.01 (− 0.06;0.04) t = − 0.5 (0.35)	− 0.12 (− 1.21;0.98) t = − 0.21 (0.39)	− 0.51^*^ (− 0.67; − 0.36) t = − 6.36 (< 0.01)			
Modern contracept. prevalence	− 1.67 (− 8.09;4.75) t = − 0.51 (0.35)	− 0.3 (− 1.2;0.6) t = − 0.65 (0.32)	− 0.01 (− 0.06;0.04) t − .39 (0.37)	0.03 (− 1.09;1.14) t = 0.05 (0.40)	− 0.5^*^ (− 0.66; − 0.33) t = − 5.84 (< 0.01)	− 0.88 (− 2.56;0.8) t = − 1.03 (0.24)		
Non-partner sexual violence	− 2.51 (− 8.86;3.84) t = − 0.77 (0.30)	− 0.24 (− 1.13;0.64) t = − 0.54 (0.35)	− 0.02 (− 0.07;0.03) t = − 0.62 (0.33)	0.05 (− 1.06;1.15) t = 0.08 (0.40)	− 0.5^*^ (− 0.66; − 0.33) t = − 5.91 (< 0.01)	− 0.97 (− 2.63;0.69) t = − 1.15 (0.21)	4.1(− 1.64;9.85) t = 1.4 (0.15)	
ART coverage	− 5.05 (− 10.73;0.63) t = − 1.74 (0.09)	0.02 (− 0.74;0.79) t = 0.06 (0.40)	− 0.02 (− 0.07;0.03) t = − 0.92 (0.26)	− 0.81 (− 1.97;0.35) t = − 1.37 (0.16)	− 0.23^*^ (− 0.46;-0.01) t = − 2.06 (0.05)	0.12 (− 1.54;1.78) t = 0.14 (0.34)	1.99 (− 3.62;7.6) t = 0.69 (0.31)	− 1.02^*^ (− 1.64; − 0.4) t = − 3.24 (< 0.01)

^*^Statistically significant at the 5% level

^a^Mean effect of a 1-unit increase in the covariate on the percent change in HIV incidence rate (95% confidence interval)

^b^All coefficients are based on a linear mixed effects model with random intercepts for country, random slopes for LDI, and log incidence rate for the outcome

^c^The model for the covariate in each row included the covariates in all the lines above. The column headings indicate the covariate to which the coefficients belong

In the LDI-only model, a 10% increase was associated with a − 17 (− 29.7; − 4.3, t = − 2.63, p < 0.01) percent decline in incidence rate, and the sum of squares of LDI was 0.17. Comparatively, when we added HIV spending, the coefficient on LDI was closer to the null, though the magnitude of the association between incidence rate and spending was lower at − 1.6 (− 2.2; − 0.9, t = − 4.87, p < 0.01) for a 10% decrease. HIV spend made a larger contribution to explaining variation in incidence rate than LDI (sum of squares = 5.5).

When we introduced knowledge scores and years of education per capita to the model, the association with the economic variables were no longer statistically significant. The latter were thus standing as partial proxies for education. Initially, a 10 unit increase in knowledge scores was associated with a 24.2 (− 33.1; − 15.2, t = − 5.3, p < 0.01) percent decline in incidence rate, but once we introduced education years, this association was no longer statistically significant. A one year increase in education years per capita was associated with a − 0.51 (− 0.67; − 0.36, t = − 6.36, p < 0.01) percent decline in incidence rates. The sum of squares for knowledge scores and education years were similar (5.31 and 5.02, respectively).

Introducing modern contraceptive prevalence [B = − 0.88 (− 2.6;0.8), t = − 1.03, p = 0.24] did not impact the coefficients on the other variables, suggesting that, for example, it does not drive the association between the economic variables and incidence rates. The sum of squares was lower than all other covariates included to that point (0.14), and it was also statistically insignificant. The magnitude of the association between modern contraceptive prevalence and incidence rate remained consistent when we added non partner sexual violence to the model. The latter was associated with a statistically insignificant percent increase in incidence rate of 4.1 (− 1.64; 9.84, t = 1.4, p = 0.15) per unit increase. Non partner sexual violence made a larger contribution than contraception to explaining incidence rate variation (sum of squares = 0.18).

Finally, adding ART rates to the model impacted the coefficients for several covariates. The associations between LDI, education years per capita, and non-partner sexual violence and incidence rates all moved closer to the null or even flipped, potentially suggesting that ART coverage operates partially as a mediator between these variables and the outcome, as well as a confounder. A 1% increase in ART coverage rate was associated with a 1.02 (− 1.64; − 0.4, t = − 3.24, p < 0.01) percent decline in incidence rate.

### Results After Covariate Selection

We first removed LDI and HIV curative care spending from the sequential model when knowledge score was added, which did not significantly affect any other coefficient. The AIC, BIC and deviance stayed nearly the same, corroborating these variables' lack of importance once we account for knowledge scores (Online Appendix Table V).

We then removed knowledge score, which had lost statistical significance when education per years capita was added. The statistical significance (p-value) and magnitude of the association between years of education and incidence rate increased in this model [B = − 0.55 (− 0.67; − 0.42 t = − 8.38 p < 0.01)] (Table 3). We also obtained nearly identical model fit statistics (Online Appendix Table V). Finally, we removed modern contraceptive prevalence and non-partner sexual violence which also resulted in similar fit statistics, confirming their lack of importance in our data (result not shown).

**Table 3:** Modelling results of the association between covariates and logged HIV incidence rate, after removing HIV spending, LDI and knowledge scores^a^

Model^c^	Years of education completed	Contraception prevalence	Non-partner sexual violence	ART coverage
Years of education completed	− 0.55^*^ (95% CI − 0.67; − 0.42) t = − 8.38 (p < 0.01)			
Contraception prevalence	− 0.5^*^ (− 0.65; − 0.35) t = − 6.65 (p < 0.01)	− 0.96 (− 2.58; 0.66) t = − 1.16 (0.2)		
Non-partner sexual violence	− 0.51^*^ (− 0.66; − 0.36) t = − 6.74 (p < 0.01)	− 1.05 (− 2.65; 0.55) t = − 1.29 (0.17)	3.86 (− 1.86; 9.59) t = 1.32 (0.17)	
ART coverage	− 0.38^*^ (− 0.56; − 0.2) t = − 4.15 (p < 0.01)	− 0.6 (− 2.16; 0.97) t = − 0.75 (0.3)	2.06 (− 3.65; 7.78) t = 0.71 (0.3)	− 0.74^*^ (− 1.3; − 0.19) t = − 2.62 (p = 0.01)

^*^Statistically significant at the 5% level

^a^Mean effect of a 1-unit increase in the covariate on the percent change in HIV incidence rate (95% confidence interval)

^b^All coefficient are based on a linear mixed effects model with random intercepts for country, random slopes for LDI, and log incidence rate for the outcome

^c^The model in each row included the covariates in all the lines above

### Final Model

The final model included education years per capita and the ART rate (Table 4), in addition to the random intercepts for country and random slopes for LDI. Each of these variables contributed to improved model fit statistics. Education years per capita contributed the most to explaining variation in incidence rates (17.43), a notable increase from its contribution in the full sequential model described above. A 1-year increase in mean education years was associated with a 0.39 (− 0.57; − 0.22, t = − 4.48 p < 0.01) % decline in the HIV incidence rate. Education was followed by ART coverage (Sum of squares = 1.1), which was associated with a 0.81 (− 1.34; − 0.28, t = − 3.01, p < 0.01) % decline in incidence rate (Table 4).

**Table 4:** Final model of the association between covariates and logged HIV incidence rate^a^

	Estimate^a^	95% confidence interval, t-value	Sum of squares^c^	F value (df = 1)^c^
(Intercept)	− 4.4^*^	(− 5.8; − 2.99) t = − 6.14 (p < 0.01)		
ART Coverage	− 0.81^*^	(− 1.34; − 0.28) t = − 3.01 (p < 0.01)	1.1	71
Years of Education Completed	− 0.39^*^	(− 0.57; − 0.22) t = − 4.48 (p < 0.01)	17.43	1136

^*^Statistically significant at the 5% level

^a^Mean effect of a 1-unit increase in the covariate on the percent change in HIV incidence rate

^b^All coefficient are based on a linear mixed effects model with random intercepts for country, random slopes for LDI, and log incidence rate for the outcome

^c^Interpretation: reduction in residual sum of squares when the covariate was added. P-values are not provided as there is no hypothesis test. Larger F-values represent greater significance for the fixed effect’s ability to explain variation in the outcome

## Discussion

After accounting for ART coverage rates and education years per capita, non-partner sexual violence, contraceptive prevalence, HIV spending, LDI and HIV knowledge scores did not contribute to explaining variation in HIV incidence rates between 2000 and 2015. Both education-related variables mediated and/or confounded the effect of HIV spending and lagged distribute income on incidence rate. Our findings underscore the importance of general education and treatment availability to address HIV incidence.

We found that improvements to education-related variables including formal education and HIV-related knowledge mattered more to explaining declines in HIV incidence rates than economic variables. Economic variables such as wealth have had mixed associations with HIV burden in Sub-Saharan Africa**.** With most conditions, health outcomes improve with wealth; poverty has similarly been assumed as a driver of HIV [27]. However, infections have skewed towards people in better socioeconomic circumstances [28], potentially because of risk factors such as the number of sexual partners [7] that also increase with wealth. Relative inequality is associated with HIV prevalence [29], which may also reflect differential access to education. Not many countries have analyzed their successes, but some countries with relatively low GDP have seen significant gains in tackling HIV. For example, Zimbabwe is not ranked highly in terms of African GDPs, yet its decline was attributed to successful campaigns for behavior change which spread through word of mouth [30].

HIV spending on care and treatment may also operate least partly through HIV knowledge and education. At the turn of the century, countries were motivated by the Millennium Development Goals which included achieving universal access to treatment and health services, and reversing the course of HIV by 2015 [31]. The developing world had large gains in ART coverage rates and strengthening of health infrastructure and systems for prevention and treatment [32]. Our findings suggest the importance of spending to reduce HIV incidence, however the association reduced once we adjusted for HIV knowledge and education. Thus, spending may operate through improvements to other socioeconomic determinants. These mechanisms can be material, for example, improving treatment access, but also qualitative as it can serve as a catalyst for more effective responses generally [33]. Despite this, compared to all health focus areas, the growth rate for global health spending on HIV reduced the most after the 2009 global financial crisis [34].

Modern contraceptive prevalence and sexual violence did not retain statistical significance in our model but are also highly linked to education. In the crude correlations, HIV knowledge scores and education both had high correlations with modern contraceptive prevalence. Contraception could reduce HIV incidence through reduced unintended HIV positive pregnancies which are also correlated with education [35]. In countries targeted by the President’s Emergency Plan for AIDS Relief, the number of unintended HIV positive births averted by contraceptive use ranged up to a maximum of 120,000 in South Africa [36]. Contraception use is also more cost-effective to prevent HIV-positive births than nevirapine [37]. Sexual violence reduces the likelihood of contraception use and increases unintended pregnancies. Female sex workers face a higher risk of sexual violence and, in this group only, hormonal oral contraception was actually found to increase susceptibility to HIV, suggesting the strong presence of unobserved risk factors [38]. In our modeling, we also found that the presence of HIV knowledge scores reduced the associations between contraception and sexual violence with HIV incidence rate, further suggesting an interplay. However, there is limited work on HIV knowledge sores in relation to these other variables to date.

### Implications

Deciding the best use of global health dollars, planning government campaigns and programs, and deciding areas of focus for on-the-ground initiatives requires strong evidence on mechanisms to reduce HIV incidence. Default thinking around infectious disease epidemics may revert to standard economic indicators, such as GDP, and consequent investment in general development. However, this study demonstrates the importance of additional nuance to HIV drivers. GDP improvements without improvements in social determinants such as education may have little effect. At the same time, investment in ART and treatment alone is not enough, and may be less effective than addressing social and structural factors such as access to education.

From a research perspective, our study contributes to understanding possible pathways between social determinants of HIV, individual behavior changes and risk factors and HIV outcomes. Analyzing a multitude of covariates’ effect on each other’s association with HIV incidence rate is a prerequisite for developing conceptual frameworks or directed acyclic graphs, an important tool for epidemiologic studies [39]. These diagrams assist in understanding exposures, outcomes, confounding, bias and, ultimately, pathways to causation. Including many covariates in a model without hypothesizing their pathways has the potential to introduce bias [10]. Blindly adjusting for different covariates can even cause the outcomes’ association with covariates already in the model to invert [11]. Evidence-based background knowledge, such as the information generated from this study, on pathways between covariates is essential for further analyses to decompose covariate effects and infer causation.

### Limitations

Our study has several limitations. First, forward selection has been extensively criticized as a method of reducing data dimensionality. However, our application and purpose renders it less problematic. The method may lead to underestimation of standard errors which can lead to falsely including or excluding variables [23]. However, with our limited sample size, there is a greater risk of over-estimating standard errors. We mitigated against this by also examining the impact on other coefficients and model fit statistics, and ensuring we understood the impact of each variable. An additional critique includes over-estimation of variance explained [22], however we mainly considered the relative explanatory power of our variables. It is however plausible that employing a different selection procedure may yield a different final model, moving away from the assumption that there is in fact ‘one true model’.

Second, we had a limited number of candidate covariates. Though this enabled us to decompose the impact of adding each of them, additional covariates could help to further understand confounders of the variables in our model. In particular, we did not account for inequality within countries, which may act as a mediator between any of the variables we included and the outcome. Considering the impact of relative inequality is a possible future direction for studies based on this research.

Third, our model could be criticized as circular because ART coverage is also used in the model that generates incidence rates. However, ART was important to include in both models. The scarcity of incidence data means that ART must be input into the model that estimates incidence. It is not possible to exclude it from this analysis either, as it is a key determinant of incidence rate and changed considerably during the 2000–2015 time period.

Future research should focus on examining different combinations of social determinants and behavioral risk factors and their effects on more epidemiological transmission parameters and demographic variables used in the HIV estimation model. This also allows adding more social determinants such as health care access and quality. Such a modeling strategy should enable us to decompose the impacts of different social determinants on the changes in HIV-related behaviors and incidence, prevalence and mortality at the country level during the last three decades.

## Conclusion

We found that ART coverage and education years per capita were important to explaining temporal variation in HIV incidence rates between 2000 and 2015 in Sub-Saharan African countries. Education reduced the effect of economic variables including LDI and HIV spending on incidence rate. Future studies can capitalize on this work to better understand the interaction pathways between the socio-behavioural and economic determinants of HIV incidence.

## Supplementary Information

Below is the link to the electronic supplementary material.

Supplementary file1 (DOCX 374 kb)

## Data Availability

Code available on request.

## Footnotes

1 See Sect. 5 of the supplementary methods appendix of the 2020 publication for details about methods and data used to generate the retrospective estimates.
