# Supplementary material for: Incidence of HIV in Sub-Saharan Africa, 2000–2015: The Interplay Between Social Determinants and Behavioral Risk Factors
Source: AIDS Behav. 2021 Jun 5;25(Suppl 2):145–54. doi: 10.1007/s10461-021-03279-9 (PMC8541936; doi:10.1007/s10461-021-03279-9)
Supplement: Supplementary file 1 — Supplementary file1 (DOCX 374 kb) [file 10461_2021_3279_MOESM1_ESM.docx]

Appendix

| **Table I:** Locations**,** surveys and years used in construction of knowledge and attitude scores | | |
| --- | --- | --- |
| **Geography** | **Series** | **Title** |
| Angola | Multiple Indicator Cluster Survey (MICS) | Angola Multiple Indicator Cluster Survey 2001 |
| Benin | Demographic and Health Survey (DHS) | Benin Demographic and Health Survey 1996 |
| Benin | Demographic and Health Survey (DHS) | Benin Demographic and Health Survey 2001 |
| Benin | Demographic and Health Survey (DHS) | Benin Demographic and Health Survey 2006 |
| Angola |  | Angola Integrated Inquiry into People's Well-Being 2008-2009 |
| Benin | Demographic and Health Survey (DHS) | Benin Demographic and Health Survey 2011-2012 |
| Benin | Multiple Indicator Cluster Survey (MICS) | Benin Multiple Indicator Cluster Survey 2014 |
| Angola | Demographic and Health Survey (DHS) | Angola Demographic and Health Survey 2015-2016 |
| Benin | Demographic and Health Survey (DHS) | Benin Demographic and Health Survey 2017-2018 |
| Botswana | Multiple Indicator Cluster Survey (MICS) | Botswana Multiple Indicator Cluster Survey 2000 |
| Botswana | Demographic and Health Survey (DHS) | Botswana Demographic and Health Survey 1988 |
| Botswana | Botswana AIDS Impact Survey | Botswana AIDS Impact Survey 2001 |
| Botswana | Botswana AIDS Impact Survey | Botswana AIDS Impact Survey 2004 |
| Botswana | Botswana AIDS Impact Survey | Botswana AIDS Impact Survey 2008 |
| Botswana | Botswana AIDS Impact Survey | Botswana AIDS Impact Survey 2013 |
| Burkina Faso | Multiple Indicator Cluster Survey (MICS) | Burkina Faso Multiple Indicator Cluster Survey 2006 |
| Burundi | Multiple Indicator Cluster Survey (MICS) | Burundi Multiple Indicator Cluster Survey 2005 |
| Burundi | Multiple Indicator Cluster Survey (MICS) | Burundi Multiple Indicator Cluster Survey 2000 |
| Burkina Faso | Demographic and Health Survey (DHS) | Burkina Faso Demographic and Health Survey 1992-1993 |
| Burkina Faso | Demographic and Health Survey (DHS) | Burkina Faso Demographic and Health Survey 1998-1999 |
| Burkina Faso | Demographic and Health Survey (DHS) | Burkina Faso Demographic and Health Survey 2003 |
| Burkina Faso | Demographic and Health Survey (DHS) | Burkina Faso Demographic and Health Survey 2010-2011 |
| Burundi | Demographic and Health Survey (DHS) | Burundi Demographic and Health Survey 2010-2011 |
| Burundi |  | Burundi HIV/AIDS Sociobehavioral Survey 2000 |
| Burundi | Demographic and Health Survey (DHS) | Burundi Demographic and Health Survey 2016-2017 |
| Burundi |  | Burundi Combined HIV/AIDS/STI Behavioral Surveillance and HIV/AIDS Seroprevalence Survey 2007 |
| Cameroon | Multiple Indicator Cluster Survey (MICS) | Cameroon Multiple Indicator Cluster Survey 2000 |
| Cameroon | Multiple Indicator Cluster Survey (MICS) | Cameroon Multiple Indicator Cluster Survey 2006 |
| Cameroon | Demographic and Health Survey (DHS) | Cameroon Demographic and Health Survey 1998 |
| Cameroon | Demographic and Health Survey (DHS) | Cameroon Demographic and Health Survey 2004 |
| Cameroon | Demographic and Health Survey (DHS) | Cameroon Demographic and Health Survey 2011 |
| Burundi |  | Burundi Sociodemographic and Reproductive Health Survey 2002 |
| Cameroon | Multiple Indicator Cluster Survey (MICS) | Cameroon Multiple Indicator Cluster Survey 2014 |
| Central African Republic | Multiple Indicator Cluster Survey (MICS) | Central African Republic Multiple Indicator Cluster Survey 2000 |
| Central African Republic | Multiple Indicator Cluster Survey (MICS) | Central African Republic Multiple Indicator Cluster Survey 2006 |
| Chad | Multiple Indicator Cluster Survey (MICS) | Chad Multiple Indicator Cluster Survey 2000 |
| Central African Republic | Demographic and Health Survey (DHS) | Central African Republic Demographic and Health Survey 1994-1995 |
| Chad | Demographic and Health Survey (DHS) | Chad Demographic and Health Survey 1996-1997 |
| Chad | Demographic and Health Survey (DHS) | Chad Demographic and Health Survey 2004 |
| Cape Verde | Demographic and Health Survey (DHS) | Cape Verde Demographic and Health Survey 2005 |
| Cape Verde | Reproductive Health Survey (RHS) | Cape Verde Reproductive Health Survey 1998 |
| Chad | Multiple Indicator Cluster Survey (MICS) | Chad Multiple Indicator Cluster Survey 2010 |
| Central African Republic | Multiple Indicator Cluster Survey (MICS)&&Demographic and Health Survey (DHS) | Central African Republic Multiple Indicator Cluster Survey 2010-2011 |
| Chad | Demographic and Health Survey (DHS) | Chad Demographic and Health Survey 2014-2015 |
| Congo | DHS AIDS Indicator Survey (AIS) | Congo AIDS Indicator Survey 2009 |
| Côte d'Ivoire | Demographic and Health Survey (DHS) | Côte d'Ivoire Demographic and Health Survey 1994 |
| Côte d'Ivoire | Demographic and Health Survey (DHS) | Côte d'Ivoire Demographic and Health Survey 1998-1999 |
| Côte d'Ivoire | Demographic and Health Survey (DHS) | Côte d'Ivoire Demographic and Health Survey 2011-2012 |
| Congo | Demographic and Health Survey (DHS) | Congo Demographic and Health Survey 2005 |
| Côte d'Ivoire | DHS AIDS Indicator Survey (AIS) | Côte d'Ivoire AIDS Indicator Survey 2005 |
| Congo | Demographic and Health Survey (DHS) | Congo Demographic and Health Survey 2011-2012 |
| Congo | Multiple Indicator Cluster Survey (MICS) | Congo Multiple Indicator Cluster Survey 2014-2015 |
| Comoros | Multiple Indicator Cluster Survey (MICS) | Comoros Multiple Indicator Cluster Survey 2000 |
| Comoros | Demographic and Health Survey (DHS) | Comoros Demographic and Health Survey 1996 |
| Comoros | Demographic and Health Survey (DHS) | Comoros Demographic and Health Survey 2012-2013 |
| Democratic Republic of the Congo | Multiple Indicator Cluster Survey (MICS) | Democratic Republic of the Congo Multiple Indicator Cluster Survey 2001 |
| Democratic Republic of the Congo | Demographic and Health Survey (DHS) | Democratic Republic of the Congo Demographic and Health Survey 2007 |
| Côte d'Ivoire | Multiple Indicator Cluster Survey (MICS) | Côte d'Ivoire Multiple Indicator Cluster Survey 2000 |
| Democratic Republic of the Congo | Multiple Indicator Cluster Survey (MICS) | Democratic Republic of the Congo Multiple Indicator Cluster Survey 2010 |
| Democratic Republic of the Congo | Demographic and Health Survey (DHS) | Democratic Republic of the Congo Demographic and Health Survey 2013-2014 |
| Côte d'Ivoire | Multiple Indicator Cluster Survey (MICS)&&Demographic and Health Survey (DHS) | Cote d'Ivoire Multiple Indicator Cluster Survey 2016 |
| Djibouti | Pan Arab Project for Family Health (PAPFAM) | Djibouti Family Health Survey 2002 |
| Djibouti | Multiple Indicator Cluster Survey (MICS) | Djibouti Multiple Indicator Cluster Survey 2006 |
| Equatorial Guinea | Multiple Indicator Cluster Survey (MICS) | Equatorial Guinea Multiple Indicator Cluster Survey 2000 |
| Eritrea | Demographic and Health Survey (DHS) | Eritrea Demographic and Health Survey 2002 |
| Eritrea | Demographic and Health Survey (DHS) | Eritrea Demographic and Health Survey 1995-1996 |
| Equatorial Guinea | Demographic and Health Survey (DHS) | Equatorial Guinea Demographic and Health Survey 2011 |
| Djibouti | Pan Arab Project for Family Health (PAPFAM) | Djibouti Family Health Survey 2012 |
| Eritrea |  | Eritrea Population and Health Survey 2010 |
| Ethiopia | Demographic and Health Survey (DHS) | Ethiopia Demographic and Health Survey 2005 |
| Ethiopia | Demographic and Health Survey (DHS) | Ethiopia Demographic and Health Survey 2000 |
| Ethiopia | Demographic and Health Survey (DHS) | Ethiopia Demographic and Health Survey 2010-2011 |
| Ethiopia | Global Fund Household Health Coverage Survey | Ethiopia Global Fund Household Health Coverage Survey 2008 |
| Ethiopia | Ethiopia Welfare Monitoring Survey | Ethiopia Welfare Monitoring Survey 2004 |
| Ethiopia | Demographic and Health Survey (DHS) | Ethiopia Demographic and Health Survey 2016 |
| The Gambia | Multiple Indicator Cluster Survey (MICS) | Gambia Multiple Indicator Cluster Survey 2000 |
| The Gambia | Multiple Indicator Cluster Survey (MICS) | Gambia Multiple Indicator Cluster Survey 2005-2006 |
| Gabon | Demographic and Health Survey (DHS) | Gabon Demographic and Health Survey 2000-2001 |
| Gabon | Demographic and Health Survey (DHS) | Gabon Demographic and Health Survey 2012 |
| The Gambia | Demographic and Health Survey (DHS) | Gambia Demographic and Health Survey 2013 |
| The Gambia | Multiple Indicator Cluster Survey (MICS) | Gambia Multiple Indicator Cluster Survey 2010 |
| Ghana | Multiple Indicator Cluster Survey (MICS)&&Demographic and Health Survey (DHS) | Ghana Multiple Indicator Cluster Survey 2006 |
| Ghana | Demographic and Health Survey (DHS) | Ghana Demographic and Health Survey 1993-1994 |
| Ghana | Demographic and Health Survey (DHS) | Ghana Demographic and Health Survey 1998-1999 |
| Ghana | Demographic and Health Survey (DHS) | Ghana Demographic and Health Survey 2003 |
| Ghana | Demographic and Health Survey (DHS)&&Child Verbal Autopsy Study (CVAS) | Ghana Demographic and Health Survey 2008 |
| Ghana | Multiple Indicator Cluster Survey (MICS)&&Demographic and Health Survey (DHS) | Ghana Multiple Indicator Cluster Survey 2011 |
| Ghana | Demographic and Health Survey (DHS) | Ghana Demographic and Health Survey 2014 |
| Guinea | Demographic and Health Survey (DHS) | Guinea Demographic and Health Survey 1992 |
| Guinea | Demographic and Health Survey (DHS) | Guinea Demographic and Health Survey 1999 |
| Guinea | Demographic and Health Survey (DHS) | Guinea Demographic and Health Survey 2005 |
| Guinea | Demographic and Health Survey (DHS) | Guinea Demographic and Health Survey 2012 |
| Guinea | Multiple Indicator Cluster Survey (MICS) | Guinea Multiple Indicator Cluster Survey 2016 |
| Guinea-Bissau | Multiple Indicator Cluster Survey (MICS) | Guinea-Bissau Multiple Indicator Cluster Survey 2000 |
| Guinea-Bissau | Multiple Indicator Cluster Survey (MICS) | Guinea-Bissau Multiple Indicator Cluster Survey 2006 |
| Guinea-Bissau | Multiple Indicator Cluster Survey (MICS) | Guinea-Bissau Multiple Indicator Cluster Survey 2010 |
| Guinea-Bissau | Multiple Indicator Cluster Survey (MICS) | Guinea-Bissau Multiple Indicator Cluster Survey 2014 |
| Kenya | Demographic and Health Survey (DHS) | Kenya Demographic and Health Survey 1993 |
| Kenya | Demographic and Health Survey (DHS) | Kenya Demographic and Health Survey 1998 |
| Kenya | Demographic and Health Survey (DHS) | Kenya Demographic and Health Survey 2003 |
| Kenya | Demographic and Health Survey (DHS) | Kenya Demographic and Health Survey 2008-2009 |
| Kenya | Kenya AIDS Indicator Survey | Kenya AIDS Indicator Survey 2007 |
| Kenya | Kenya AIDS Indicator Survey | Kenya AIDS Indicator Survey 2012-2013 |
| Kenya | Demographic and Health Survey (DHS) | Kenya Demographic and Health Survey 2014 |
| Kenya | Kenya Integrated Household Budget Survey | Kenya Integrated Household Budget Survey 2005-2006 |
| Kenya | Multiple Indicator Cluster Survey (MICS) | Kenya Multiple Indicator Cluster Survey 2000 |
| Lesotho | Demographic and Health Survey (DHS) | Lesotho Demographic and Health Survey 2004-2005 |
| Lesotho | Demographic and Health Survey (DHS) | Lesotho Demographic and Health Survey 2009-2010 |
| Lesotho | Demographic and Health Survey (DHS) | Lesotho Demographic and Health Survey 2014 |
| Lesotho | Multiple Indicator Cluster Survey (MICS) | Lesotho Multiple Indicator Cluster Survey 2000 |
| Liberia | Demographic and Health Survey (DHS) | Liberia Demographic and Health Survey 2006-2007 |
| Liberia | Demographic and Health Survey (DHS) | Liberia Demographic and Health Survey 2013 |
| Lesotho |  | Lesotho Reproductive Health Survey 2002 |
| Lesotho | Population-Based HIV Impact Assessments (PHIA) | Lesotho Population-Based HIV Impact Assessment 2016-2017 |
| Malawi | Demographic and Health Survey (DHS) | Malawi Demographic and Health Survey 1992 |
| Malawi | Demographic and Health Survey (DHS) | Malawi Demographic and Health Survey 2000 |
| Malawi | Demographic and Health Survey (DHS) | Malawi Demographic and Health Survey 2004-2005 |
| Malawi | Demographic and Health Survey (DHS) | Malawi Demographic and Health Survey 2010 |
| Malawi | Demographic and Health Survey (DHS) | Malawi Demographic and Health Survey 2015-2016 |
| Madagascar | Demographic and Health Survey (DHS) | Madagascar Demographic and Health Survey 1997 |
| Madagascar | Demographic and Health Survey (DHS) | Madagascar Demographic and Health Survey 2003-2004 |
| Madagascar | Demographic and Health Survey (DHS) | Madagascar Demographic and Health Survey 2008-2009 |
| Madagascar | Multiple Indicator Cluster Survey (MICS) | Madagascar Multiple Indicator Cluster Survey 2000 |
| Madagascar | | Madagascar STD/AIDS Behavioral Surveillance Survey 2004 |
| Malawi | Multiple Indicator Cluster Survey (MICS) | Malawi Multiple Indicator Cluster Survey 2006 |
| Malawi | DHS Knowledge, Attitudes and Practices in Health Survey (KAP) | Malawi Knowledge, Attitudes and Practices in Health Survey 1996 |
| Malawi | Malawi Welfare Monitoring Survey | Malawi Welfare Monitoring Survey 2011-2012 |
| Malawi | Multiple Indicator Cluster Survey (MICS) | Malawi Multiple Indicator Cluster Survey 2013-2014 |
| Malawi | Population-Based HIV Impact Assessments (PHIA) | Malawi Population-Based HIV Impact Assessment 2015-2016 |
| Malawi | Malawi Welfare Monitoring Survey | Malawi Welfare Monitoring Survey 2014-2015 |
| Mali | Demographic and Health Survey (DHS) | Mali Demographic and Health Survey 2006 |
| Mali | Demographic and Health Survey (DHS) | Mali Demographic and Health Survey 1995-1996 |
| Mali | Demographic and Health Survey (DHS) | Mali Demographic and Health Survey 2001 |
| Mali | Demographic and Health Survey (DHS) | Mali Demographic and Health Survey 2012-2013 |
| Mali | Multiple Indicator Cluster Survey (MICS) | Mali Multiple Indicator Cluster Survey 2015 |
| Mali | Multiple Indicator Cluster Survey (MICS) | Mali Multiple Indicator Cluster Survey 2009-2010 |
| Mauritania | Demographic and Health Survey (DHS) | Mauritania Demographic and Health Survey 2000-2001 |
| Mozambique | DHS AIDS Indicator Survey (AIS) | Mozambique AIDS Indicator Survey 2009 |
| Mozambique | Demographic and Health Survey (DHS) | Mozambique Demographic and Health Survey 1997 |
| Mozambique | Demographic and Health Survey (DHS) | Mozambique Demographic and Health Survey 2003-2004 |
| Mozambique | Multiple Indicator Cluster Survey (MICS) | Mozambique Multiple Indicator Cluster Survey 2008-2009 |
| Mozambique | Demographic and Health Survey (DHS) | Mozambique Demographic and Health Survey 2011 |
| Mozambique | DHS AIDS Indicator Survey (AIS) | Mozambique AIDS Indicator Survey 2015 |
| Mauritania | Multiple Indicator Cluster Survey (MICS) | Mauritania Multiple Indicator Cluster Survey 2007 |
| Mauritania | Multiple Indicator Cluster Survey (MICS) | Mauritania Multiple Indicator Cluster Survey 2011 |
| Mauritania | Multiple Indicator Cluster Survey (MICS) | Mauritania Multiple Indicator Cluster Survey 2015 |
| Namibia | Demographic and Health Survey (DHS) | Namibia Demographic and Health Survey 2000 |
| Namibia | Demographic and Health Survey (DHS) | Namibia Demographic and Health Survey 2006-2007 |
| Niger | Demographic and Health Survey (DHS) | Niger Demographic and Health Survey 2006 |
| Niger | Demographic and Health Survey (DHS) | Niger Demographic and Health Survey 1998 |
| Niger | Demographic and Health Survey (DHS) | Niger Demographic and Health Survey 2012 |
| Namibia | Demographic and Health Survey (DHS) | Namibia Demographic and Health Survey 2013 |
| Niger | Demographic and Health Survey (DHS) | Niger Demographic and Health Survey 2017 |
| Namibia | Population-Based HIV Impact Assessments (PHIA) | Namibia Population-Based HIV Impact Assessment 2017 |
| Niger | Multiple Indicator Cluster Survey (MICS) | Niger Multiple Indicator Cluster Survey 2000 |
| Nigeria | Demographic and Health Survey (DHS) | Nigeria Demographic and Health Survey 2003 |
| Nigeria | Demographic and Health Survey (DHS) | Nigeria Demographic and Health Survey 2008 |
| Nigeria | Demographic and Health Survey (DHS) | Nigeria Demographic and Health Survey 2013 |
| Nigeria | Multiple Indicator Cluster Survey (MICS) | Nigeria Multiple Indicator Cluster Survey 2007 |
| Nigeria | Nigeria Living Standards Survey | Nigeria Living Standards Survey 2003-2004 |
| Nigeria | Multiple Indicator Cluster Survey (MICS) | Nigeria Multiple Indicator Cluster Survey 2011 |
| Nigeria | Nigeria National HIV/AIDS and Reproductive Health Survey | Nigeria National HIV/AIDS & Reproductive Health Survey 2003 |
| Nigeria | Nigeria Living Standards Survey | Nigeria Living Standards Survey 2008-2010 |
| Nigeria | Nigeria National HIV/AIDS and Reproductive Health Survey | Nigeria National HIV/AIDS and Reproductive Health Survey 2005 |
| Nigeria | Multiple Indicator Cluster Survey (MICS) | Nigeria Multiple Indicator Cluster Survey with National Immunization Coverage Survey Supplement 2016-2017 |
| Nigeria | Nigeria National HIV/AIDS and Reproductive Health Survey | Nigeria National HIV/AIDS and Reproductive Health Survey 2012 |
| Nigeria | Nigeria National HIV/AIDS and Reproductive Health Survey | Nigeria National HIV/AIDS and Reproductive Health Survey 2007 |
| Rwanda | Demographic and Health Survey (DHS) | Rwanda Demographic and Health Survey 2000 |
| Rwanda | Demographic and Health Survey (DHS) | Rwanda Demographic and Health Survey 2005 |
| Rwanda | Demographic and Health Survey (DHS) | Rwanda Demographic and Health Survey 2010-2011 |
| Rwanda | DHS Special Demographic and Health Survey (Special DHS) | Rwanda Special Demographic and Health Survey 2011 |
| Rwanda | Demographic and Health Survey (DHS) | Rwanda Demographic and Health Survey 2014-2015 |
| Rwanda |  | Rwanda AIDS Indicator and HIV Incidence Survey 2013 |
| Senegal | Demographic and Health Survey (DHS) | Senegal Demographic and Health Survey 1992-1993 |
| Senegal | Demographic and Health Survey (DHS) | Senegal Demographic and Health Survey 1997 |
| Senegal | Demographic and Health Survey (DHS) | Senegal Demographic and Health Survey 1999-2000 |
| Senegal | Demographic and Health Survey (DHS) | Senegal Demographic and Health Survey 2005 |
| Senegal | Multiple Indicator Cluster Survey (MICS) | Senegal Multiple Indicator Cluster Survey 2000 |
| Senegal | Demographic and Health Survey (DHS) | Senegal Demographic and Health Survey 2010-2011 |
| Senegal | Demographic and Health Survey (DHS) | Senegal Continuous Demographic and Health Survey 2014 |
| Senegal | Demographic and Health Survey (DHS) | Senegal Continuous Demographic and Health Survey 2015 |
| Senegal | Demographic and Health Survey (DHS) | Senegal Continuous Demographic and Health Survey 2016 |
| Senegal | Demographic and Health Survey (DHS) | Senegal Continuous Demographic and Health Survey 2017 |
| Sao Tome and Principe | Multiple Indicator Cluster Survey (MICS) | Sao Tome and Principe Multiple Indicator Cluster Survey 2006 |
| Sao Tome and Principe | Demographic and Health Survey (DHS) | Sao Tome and Principe Demographic and Health Survey 2008-2009 |
| Sao Tome and Principe | Multiple Indicator Cluster Survey (MICS) | Sao Tome and Principe Multiple Indicator Cluster Survey 2000 |
| Sao Tome and Principe | Multiple Indicator Cluster Survey (MICS)&&Demographic and Health Survey (DHS) | Sao Tome and Principe Multiple Indicator Cluster Survey 2014 |
| Sierra Leone | Multiple Indicator Cluster Survey (MICS) | Sierra Leone Multiple Indicator Cluster Survey 2000 |
| Sierra Leone | Multiple Indicator Cluster Survey (MICS) | Sierra Leone Multiple Indicator Cluster Survey 2005 |
| Sierra Leone | Demographic and Health Survey (DHS) | Sierra Leone Demographic and Health Survey 2008 |
| Sierra Leone | Multiple Indicator Cluster Survey (MICS) | Sierra Leone Multiple Indicator Cluster Survey 2010 |
| Sierra Leone | Demographic and Health Survey (DHS) | Sierra Leone Demographic and Health Survey 2013 |
| Sierra Leone | Multiple Indicator Cluster Survey (MICS) | Sierra Leone Multiple Indicator Cluster Survey 2017 |
| Sierra Leone | | Sierra Leone National Population-Based HIV Seroprevalence Survey 2005 |
| Sierra Leone | | Sierra Leone HIV/AIDS Seroprevalence and Behavioral Risk Factor Survey 2002 |
| Somalia | Multiple Indicator Cluster Survey (MICS) | Somalia Multiple Indicator Cluster Survey 1999 |
| Somalia | Multiple Indicator Cluster Survey (MICS) | Somalia Multiple Indicator Cluster Survey 2006 |
| South Africa | Demographic and Health Survey (DHS) | South Africa Demographic and Health Survey 1998 |
| South Africa | South Africa National HIV Prevalence, Incidence, Behavior and Communication Survey (SABSSM) | South Africa HIV/AIDS Behavioral Risks, Sero-Status, and Mass Media Impact Survey 2002 |
| South Africa | Demographic and Health Survey (DHS) | South Africa Demographic and Health Survey 2003-2004 |
| South Africa | Demographic and Health Survey (DHS) | South Africa Demographic and Health Survey 2016 |
| South Africa | South Africa National HIV Prevalence, Incidence, Behavior and Communication Survey (SABSSM) | South Africa National HIV Prevalence, Incidence, Behavior and Communication Survey 2008-2009 |
| South Africa | South Africa National HIV Prevalence, Incidence, Behavior and Communication Survey (SABSSM) | South Africa National HIV Prevalence, Incidence, Behavior and Communication Survey 2004-2005 |
| South Africa | South Africa National HIV Prevalence, Incidence, Behavior and Communication Survey (SABSSM) | South Africa National HIV Prevalence, Incidence, and Behavior Survey 2011-2012 |
| South Africa | South Africa National HIV Prevalence, Incidence, Behavior and Communication Survey (SABSSM) | South Africa National HIV Prevalence, Incidence, Behavior and Communication Survey 2017 |
| Sudan | Multiple Indicator Cluster Survey (MICS) | Sudan - North Multiple Indicator Cluster Survey 2010 |
| Sudan | Multiple Indicator Cluster Survey (MICS) | Sudan - South Multiple Indicator Cluster Survey 2010 |
| South Sudan | | South Sudan Lot Quality Assurance Sampling Survey 2011 |
| South Sudan | | South Sudan Lot Quality Assurance Sampling Survey 2015 |
| Swaziland | Reproductive Health Survey (RHS) | Swaziland Family Planning/Maternal and Child Health Survey 1988-1989 |
| Swaziland | Multiple Indicator Cluster Survey (MICS) | Swaziland Multiple Indicator Cluster Survey 2000 |
| Swaziland | Demographic and Health Survey (DHS) | Swaziland Demographic and Health Survey 2006-2007 |
| Swaziland | Multiple Indicator Cluster Survey (MICS) | Swaziland Multiple Indicator Cluster Survey 2010 |
| Swaziland | Multiple Indicator Cluster Survey (MICS) | Swaziland Multiple Indicator Cluster Survey 2014 |
| Sudan | Multiple Indicator Cluster Survey (MICS) | Sudan Multiple Indicator Cluster Survey in the Southern Areas 1999 |
| Sudan | Multiple Indicator Cluster Survey (MICS) | Sudan Multiple Indicator Cluster Survey 2000 |
| Sudan | Pan Arab Project for Family Health (PAPFAM) | Sudan Family Health Survey 2006 |
| Sudan | Multiple Indicator Cluster Survey (MICS) | Sudan Multiple Indicator Cluster Survey 2014 |
| Tanzania | DHS AIDS Indicator Survey (AIS) | Tanzania AIDS Indicator Survey 2003-2004 |
| Tanzania | DHS AIDS Indicator Survey (AIS) | Tanzania HIV/AIDS and Malaria Indicator Survey 2007-2008 |
| Tanzania | Demographic and Health Survey (DHS) | Tanzania Demographic and Health Survey 1991-1992 |
| Tanzania | Demographic and Health Survey (DHS) | Tanzania Demographic and Health Survey 1996 |
| Tanzania | Demographic and Health Survey (DHS) | Tanzania Demographic and Health Survey 1999 |
| Tanzania | Demographic and Health Survey (DHS) | Tanzania Demographic and Health Survey 2004-2005 |
| Tanzania | Demographic and Health Survey (DHS) | Tanzania Demographic and Health Survey 2009-2010 |
| Tanzania | DHS AIDS Indicator Survey (AIS) | Tanzania AIDS Indicator Survey 2011-2012 |
| Tanzania | DHS Knowledge, Attitudes and Practices in Health Survey (KAP) | Tanzania Knowledge, Attitudes, and Practices Survey 1994 |
| Tanzania | Population-Based HIV Impact Assessments (PHIA) | Tanzania HIV Impact Survey 2016-2017 |
| Togo | Multiple Indicator Cluster Survey (MICS) | Togo Multiple Indicator Cluster Survey 2000 |
| Togo | Multiple Indicator Cluster Survey (MICS) | Togo Multiple Indicator Cluster Survey 2006 |
| Togo | Demographic and Health Survey (DHS) | Togo Demographic and Health Survey 1998 |
| Togo | Multiple Indicator Cluster Survey (MICS) | Togo Multiple Indicator Cluster Survey 2010 |
| Togo | Demographic and Health Survey (DHS) | Togo Demographic and Health Survey 2013-2014 |
| Uganda | DHS AIDS Indicator Survey (AIS) | Uganda AIDS Indicator Survey 2004-2005 |
| Uganda | Demographic and Health Survey (DHS) | Uganda Demographic and Health Survey 1995 |
| Uganda | Demographic and Health Survey (DHS) | Uganda Demographic and Health Survey 2000-2001 |
| Uganda | Demographic and Health Survey (DHS) | Uganda Demographic and Health Survey 2006 |
| Uganda | DHS AIDS Indicator Survey (AIS) | Uganda AIDS Indicator Survey 2011 |
| Uganda | Demographic and Health Survey (DHS) | Uganda Demographic and Health Survey 2011 |
| Uganda |  | Uganda Lot Quality Assurance Sampling Survey 2004 |
| Uganda | Demographic and Health Survey (DHS) | Uganda Demographic and Health Survey 2016 |
| Uganda |  | Uganda Lot Quality Assurance Sampling Survey 2005 |
| Uganda |  | Uganda Lot Quality Assurance Sampling Survey 2006 |
| Tanzania | Population-Based HIV Impact Assessments (PHIA) | Uganda Population-Based HIV Impact Assessment 2016-2017 |
| Zambia | Demographic and Health Survey (DHS) | Zambia Demographic and Health Survey 1992 |
| Zambia | Demographic and Health Survey (DHS) | Zambia Demographic and Health Survey 1996-1997 |
| Zambia | Demographic and Health Survey (DHS) | Zambia Demographic and Health Survey 2001-2002 |
| Zambia | Demographic and Health Survey (DHS) | Zambia Demographic and Health Survey 2007 |
| Zambia | Global Fund Household Health Coverage Survey | Zambia Global Fund Household Health Coverage Survey 2008 |
| Zambia | Demographic and Health Survey (DHS) | Zambia Demographic and Health Survey 2013-2014 |
| Zambia | Multiple Indicator Cluster Survey (MICS) | Zambia Multiple Indicator Cluster Survey 1999 |
| Zimbabwe | Demographic and Health Survey (DHS) | Zimbabwe Demographic and Health Survey 1988-1989 |
| Zimbabwe | Demographic and Health Survey (DHS) | Zimbabwe Demographic and Health Survey 1994 |
| Zimbabwe | Demographic and Health Survey (DHS) | Zimbabwe Demographic and Health Survey 1999 |
| Zimbabwe | Demographic and Health Survey (DHS) | Zimbabwe Demographic and Health Survey 2005-2006 |
| Zambia | Zambia Sexual Behavior Survey | Zambia Sexual Behavior Survey 1998 |
| Zambia | Zambia Sexual Behavior Survey | Zambia Sexual Behavior Survey 2000 |
| Zambia | Zambia Sexual Behavior Survey | Zambia Sexual Behavior Survey 2003 |
| Zambia | Zambia Sexual Behavior Survey | Zambia Sexual Behavior Survey 2005 |
| Zimbabwe | Demographic and Health Survey (DHS) | Zimbabwe Demographic and Health Survey 2010-2011 |
| Zambia | Zambia Sexual Behavior Survey | Zambia Sexual Behavior Survey 2009 |
| Zimbabwe | Demographic and Health Survey (DHS) | Zimbabwe Demographic and Health Survey 2015 |
| Zambia | Population-Based HIV Impact Assessments (PHIA) | Zambia Population-Based HIV Impact Assessment 2016 |
| Zimbabwe | Multiple Indicator Cluster Survey (MICS) | Zimbabwe Multiple Indicator Monitoring Survey 2009 |
| Zimbabwe |  | Zimbabwe National Maternal and Child Health Family Planning Survey 1997 |
| Zimbabwe | Multiple Indicator Cluster Survey (MICS) | Zimbabwe Multiple Indicator Cluster Survey 2014 |
| Zimbabwe | Population-Based HIV Impact Assessments (PHIA) | Zimbabwe Population-Based HIV Impact Assessment 2015-2016 |

**Figure 1:** Incidence rate over time for 46 countries in Sub-Saharan Africa, and overall mean


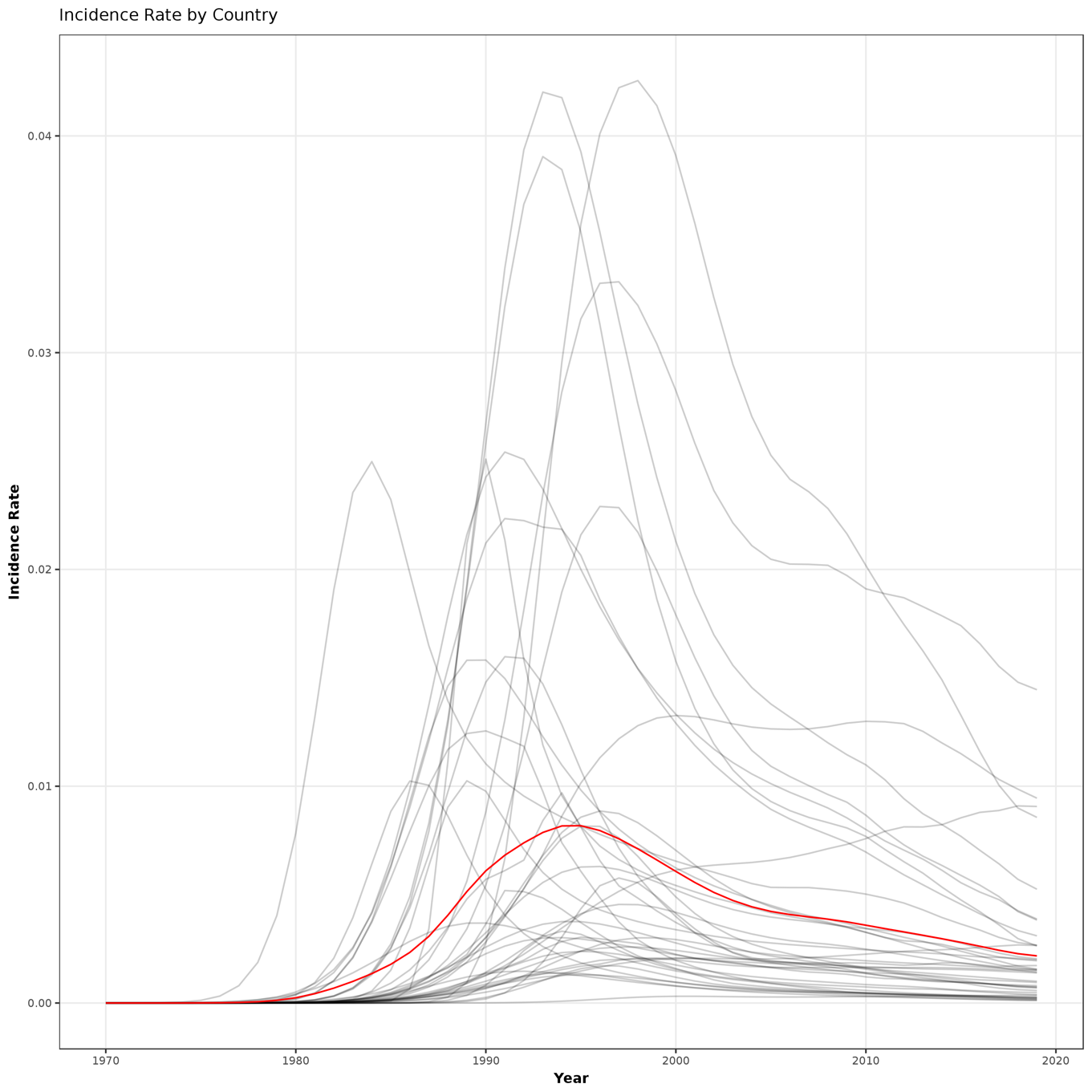


| **Table II**: All data sources |  |  |
| --- | --- | --- |
| Location | Number of Prevalence Surveys (Year range) | ANC Site Years (Year range) |
| Angola | 1 (2015) | 192 (2004-2013) |
| Benin | 1 (2006-2012) | 886 (1990-2017) |
| Botswana | 3 (2004-2013) | 244 (1991-2011) |
| Burkina Faso | 2 (2003-2010) | 233 (1987-2017) |
| Burundi | 3 (2002-2016) | 92 (1999-2015) |
| Cameroon | 3 (2004-2017) | 307 (1988-2016) |
| Cape Verde | 1 (2005) | 31 (1989-2013) |
| Central African Republic | 1 (2010) | 118 (1989-2015) |
| Chad | 1 (2015) | 94 (1992-2014) |
| Congo (Brazzaville) | 1 (2009) | 213 (1990-2015) |
| Côte d'Ivoire | 3 (2005-2017) | 180 (2000-2017) |
| DR Congo | 2 (2007-2013) | 360 (1985-2015) |
| Djibouti | 1 (2002) | 111 (2002-2014) |
| Dominican Republic | 3 (2002-2013) | 334 (1991-2013) |
| Equatorial Guinea | 1 (2004-2011) | 14 (1996-2012) |
| Eritrea | 1 (2010) | 122 (1994-2018) |
| Ethiopia | 4 (2005-2018) | 746 (1989-2016) |
| Gabon | 1 (2012) | 48 (2000-2009) |
| Ghana | 2 (2003-2014) | 756 (1994-2017) |
| Guinea | 2 (2005-2012) | 77 (1990-2015) |
| Haiti | 2 (2006-2012) | 171 (1993-2015) |
| Kenya | 4 (2003-2013) | 307 (1990-2011) |
| Lesotho | 4 (2004-2016) | 108 (1991-2016) |
| Liberia | 2 (2007-2013) | 126 (2006-2017) |
| Malawi | 3 (2004-2016) | 238 (1994-2010) |
| Mali | 3 (2001-2012) | 103 (2002-2012) |
| Mozambique | 2 (2009-2015) | 239 (1988-2011) |
| Namibia | 2 (2013-2017) | 317 (1992-2016) |
| Niger | 3 (2002-2012) | 119 (1990-2014) |
| Nigeria | 1 (2018) | 968 (1992-2014) |
| Rwanda | 4 (2005-2015) | 169 (1998-2013) |
| Senegal | 3 (2005-2017) | 146 (1989-2014) |
| Sierra Leone | 3 (2002-2013) | 88 (2003-2015) |
| South Africa | 4 (2005-2017) | 823 (1990-2014) |
| Tanzania | 4 (2004-2016) | 657 (1986-2016) |
| The Gambia | 1 (2013) | 110 (1995-2016) |
| Togo | 1 (2014) | 488 (1994-2016) |
| Uganda | 3 (2005-2016) | 430 (1986-2014) |
| Zambia | 4 (2002-2016) | 162 (1993-2011) |
| Zimbabwe | 4 (2005-2016) | 162 (2000-2012) |
| eSwatini | 2 (2006-2016) | 100 (1994-2010) |

**Table III** Covariate values by country (2000, 2015) and percent changes

| **Table IIIa:** ART coverage rate by country | | | |
| --- | --- | --- | --- |
| Country | 2000 | 2015 |  |
| Angola | 0 | 0.23 |  |
| Burundi | 0 | 0.43 |  |
| Benin | 0 | 0.46 |  |
| Burkina Faso | 0 | 0.46 |  |
| Botswana | 0 | 0.71 |  |
| Central African Republic | 0 | 0.22 |  |
| Côte d'Ivoire | 0 | 0.32 |  |
| Cameroon | 0 | 0.26 |  |
| DR Congo | 0 | 0.28 |  |
| Congo (Brazzaville) | 0 | 0.25 |  |
| Comoros | 0 | 0.69 |  |
| Cape Verde | 0 | 0.51 |  |
| Djibouti | 0 | 0.14 |  |
| Eritrea | 0 | 0.32 |  |
| Ethiopia | 0 | 0.44 |  |
| Gabon | 0 | 0.53 |  |
| Ghana | 0 | 0.28 |  |
| Guinea | 0 | 0.31 |  |
| The Gambia | 0 | 0.21 |  |
| Guinea-Bissau | 0 | 0.31 |  |
| Equatorial Guinea | 0 | 0.24 |  |
| Kenya | 0 | 0.49 |  |
| Liberia | 0 | 0.19 |  |
| Lesotho | 0 | 0.41 |  |
| Madagascar | 0 | 0.03 |  |
| Mali | 0 | 0.31 |  |
| Mozambique | 0 | 0.34 |  |
| Mauritania | 0 | 0.82 |  |
| Malawi | 0 | 0.59 |  |
| Namibia | 0 | 0.68 |  |
| Niger | 0 | 0.3 |  |
| Nigeria | 0 | 0.41 |  |
| Rwanda | 0 | 0.73 |  |
| Senegal | 0 | 0.35 |  |
| Sierra Leone | 0 | 0.18 |  |
| Somalia | 0 | 0.05 |  |
| South Sudan | 0 | 0.16 |  |
| São Tomé and Príncipe | 0 | 0.86 |  |
| eSwatini | 0 | 0.59 |  |
| Chad | 0 | 0.42 |  |
| Togo | 0 | 0.34 |  |
| Tanzania | 0 | 0.47 |  |
| Uganda | 0 | 0.54 |  |
| South Africa | 0 | 0.46 |  |
| Zambia | 0 | 0.62 |  |
| Zimbabwe | 0 | 0.64 |  |

| **Table IIIb:** HIV Attitude Score by country | | | |
| --- | --- | --- | --- |
| Country | 2000 | 2015 | Percent Change |
| Angola | 0.52 | 0.68 | 30.08 |
| Burundi | 0.68 | 0.83 | 21.37 |
| Benin | 0.37 | 0.51 | 38.44 |
| Burkina Faso | 0.5 | 0.56 | 12.5 |
| Botswana | 0.65 | 0.83 | 27.74 |
| Central African Republic | 0.6 | 0.71 | 19.36 |
| Côte d'Ivoire | 0.53 | 0.62 | 16.36 |
| Cameroon | 0.44 | 0.64 | 46.42 |
| DR Congo | 0.48 | 0.53 | 8.69 |
| Congo (Brazzaville) | 0.51 | 0.63 | 24.92 |
| Comoros | 0.43 | 0.49 | 13.25 |
| Cape Verde | 0.57 | 0.74 | 30.38 |
| Djibouti | 0.57 | 0.59 | 3.09 |
| Eritrea | 0.56 | 0.73 | 29.97 |
| Ethiopia | 0.41 | 0.67 | 63.46 |
| Gabon | 0.62 | 0.72 | 16.36 |
| Ghana | 0.49 | 0.54 | 10.87 |
| Guinea | 0.41 | 0.51 | 23.23 |
| The Gambia | 0.44 | 0.58 | 31.07 |
| Guinea-Bissau | 0.43 | 0.57 | 30.93 |
| Equatorial Guinea | 0.48 | 0.64 | 34.06 |
| Kenya | 0.65 | 0.8 | 21.79 |
| Liberia | 0.53 | 0.53 | 0.58 |
| Lesotho | 0.6 | 0.8 | 32.53 |
| Madagascar | 0.4 | 0.51 | 29.07 |
| Mali | 0.49 | 0.59 | 20.24 |
| Mozambique | 0.51 | 0.68 | 33.99 |
| Mauritania | 0.47 | 0.49 | 4.68 |
| Malawi | 0.68 | 0.74 | 9.14 |
| Namibia | 0.67 | 0.77 | 14.92 |
| Niger | 0.39 | 0.49 | 26.12 |
| Nigeria | 0.36 | 0.55 | 51.3 |
| Rwanda | 0.61 | 0.87 | 41.77 |
| Senegal | 0.44 | 0.49 | 11.83 |
| Sierra Leone | 0.38 | 0.5 | 31.29 |
| Somalia | 0.43 | 0.45 | 4.02 |
| South Sudan | 0.54 | 0.51 | -6.07 |
| São Tomé and Príncipe | 0.55 | 0.66 | 19.92 |
| eSwatini | 0.72 | 0.82 | 14.27 |
| Chad | 0.49 | 0.6 | 22.15 |
| Togo | 0.45 | 0.64 | 40.77 |
| Tanzania | 0.64 | 0.75 | 16.9 |
| Uganda | 0.61 | 0.71 | 16.36 |
| South Africa | 0.72 | 0.68 | -5.44 |
| Zambia | 0.61 | 0.74 | 20.72 |
| Zimbabwe | 0.62 | 0.8 | 28.8 |

| **Table IIIc:** Modern Contraceptive Prevalence by country | | | |
| --- | --- | --- | --- |
| Country | 2000 | 2015 | Percent Change |
| Angola | 0.03 | 0.12 | 267.21 |
| Burundi | 0.06 | 0.12 | 108.86 |
| Benin | 0.06 | 0.1 | 73.85 |
| Burkina Faso | 0.07 | 0.22 | 191.81 |
| Botswana | 0.37 | 0.55 | 48.36 |
| Central African Republic | 0.05 | 0.09 | 94.35 |
| Côte d'Ivoire | 0.08 | 0.14 | 69.4 |
| Cameroon | 0.07 | 0.21 | 213.6 |
| DR Congo | 0.04 | 0.12 | 240.72 |
| Congo (Brazzaville) | 0.11 | 0.24 | 107.85 |
| Comoros | 0.13 | 0.13 | 0.75 |
| Cape Verde | 0.47 | 0.47 | -0.2 |
| Djibouti | 0.06 | 0.17 | 202.51 |
| Eritrea | 0.04 | 0.08 | 112.76 |
| Ethiopia | 0.05 | 0.24 | 401.22 |
| Gabon | 0.14 | 0.25 | 80.78 |
| Ghana | 0.12 | 0.18 | 53.42 |
| Guinea | 0.05 | 0.07 | 47.97 |
| The Gambia | 0.06 | 0.07 | 20.56 |
| Guinea-Bissau | 0.04 | 0.23 | 493.78 |
| Equatorial Guinea | 0.04 | 0.12 | 170.68 |
| Kenya | 0.28 | 0.38 | 33.87 |
| Liberia | 0.08 | 0.21 | 157.34 |
| Lesotho | 0.22 | 0.49 | 127.14 |
| Madagascar | 0.09 | 0.2 | 127.9 |
| Mali | 0.06 | 0.1 | 74.98 |
| Mozambique | 0.09 | 0.15 | 56.66 |
| Mauritania | 0.03 | 0.05 | 46.38 |
| Malawi | 0.2 | 0.44 | 118.76 |
| Namibia | 0.37 | 0.51 | 40.11 |
| Niger | 0.04 | 0.12 | 246.83 |
| Nigeria | 0.07 | 0.12 | 68.73 |
| Rwanda | 0.03 | 0.26 | 685.88 |
| Senegal | 0.06 | 0.15 | 147.92 |
| Sierra Leone | 0.04 | 0.2 | 370.77 |
| Somalia | 0.02 | 0.03 | 73.18 |
| South Sudan | 0.01 | 0.02 | 54.42 |
| São Tomé and Príncipe | 0.18 | 0.31 | 70.59 |
| eSwatini | 0.2 | 0.51 | 153.82 |
| Chad | 0.01 | 0.04 | 203.69 |
| Togo | 0.15 | 0.17 | 14.77 |
| Tanzania | 0.15 | 0.27 | 72.09 |
| Uganda | 0.13 | 0.24 | 83.67 |
| South Africa | 0.48 | 0.6 | 23.49 |
| Zambia | 0.15 | 0.32 | 107.81 |
| Zimbabwe | 0.35 | 0.48 | 34.69 |

| **Table IIId:** Education Years per Capita by country | | | |
| --- | --- | --- | --- |
| Country | 2000 | 2015 | Percent Change |
| Angola | 4.08 | 6.36 | 56.09 |
| Burundi | 3.59 | 4.69 | 30.54 |
| Benin | 3 | 4.92 | 63.96 |
| Burkina Faso | 1.59 | 2.84 | 78.73 |
| Botswana | 7.63 | 9.73 | 27.41 |
| Central African Republic | 3.74 | 5.79 | 54.86 |
| Côte d'Ivoire | 3.98 | 5.23 | 31.44 |
| Cameroon | 6.05 | 7.99 | 32.14 |
| DR Congo | 6.17 | 7.58 | 22.83 |
| Congo (Brazzaville) | 7.07 | 8.95 | 26.52 |
| Comoros | 4.42 | 7.08 | 60.18 |
| Cape Verde | 4.81 | 6.89 | 43.14 |
| Djibouti | 4.58 | 6.65 | 45.25 |
| Eritrea | 3.84 | 6.49 | 69.13 |
| Ethiopia | 2.17 | 4.02 | 85.1 |
| Gabon | 7.21 | 9.06 | 25.58 |
| Ghana | 6.6 | 7.84 | 18.8 |
| Guinea | 2.44 | 4.14 | 70.09 |
| The Gambia | 4.07 | 5.93 | 45.91 |
| Guinea-Bissau | 2.63 | 4.46 | 69.5 |
| Equatorial Guinea | 6.23 | 8.69 | 39.46 |
| Kenya | 7.26 | 8.76 | 20.73 |
| Liberia | 4.2 | 5.72 | 36.18 |
| Lesotho | 6.3 | 7.86 | 24.66 |
| Madagascar | 4.32 | 5.19 | 20.17 |
| Mali | 1.71 | 2.89 | 69.22 |
| Mozambique | 2.92 | 5 | 71.23 |
| Mauritania | 4.38 | 6.34 | 44.59 |
| Malawi | 5 | 6.68 | 33.68 |
| Namibia | 7.27 | 9.16 | 25.98 |
| Niger | 1.9 | 2.77 | 45.65 |
| Nigeria | 5.85 | 7.54 | 28.96 |
| Rwanda | 3.89 | 5.35 | 37.6 |
| Senegal | 3.07 | 4.16 | 35.47 |
| Sierra Leone | 2.71 | 4.77 | 76.26 |
| Somalia | 2.73 | 4.31 | 58.05 |
| South Sudan | 1.34 | 2.32 | 73.25 |
| São Tomé and Príncipe | 4.65 | 6.94 | 49.14 |
| eSwatini | 7.11 | 8.58 | 20.68 |
| Chad | 2.31 | 3.56 | 53.71 |
| Togo | 4.2 | 6.31 | 50.52 |
| Tanzania | 5.31 | 6.49 | 22.2 |
| Uganda | 5.25 | 6.93 | 32.13 |
| South Africa | 9.04 | 10.85 | 20.02 |
| Zambia | 6.6 | 7.88 | 19.31 |
| Zimbabwe | 8.2 | 9.66 | 17.72 |

| **Table IIIe:** HIV Curative Care Spending by country | | | |
| --- | --- | --- | --- |
| Country | 2000 | 2015 | Percent Change |
| Angola | 0.04 | 0.7 | 1766.77 |
| Burundi | 0.15 | 0.47 | 210.43 |
| Benin | 0.15 | 0.45 | 207.4 |
| Burkina Faso | 0.22 | 0.58 | 162.57 |
| Botswana | 41 | 49.88 | 21.67 |
| Central African Republic | 0.1 | 0.05 | -45.94 |
| Côte d'Ivoire | 0.38 | 1.89 | 403.05 |
| Cameroon | 0.28 | 1.17 | 322.03 |
| DR Congo | 0.21 | 0.8 | 278.21 |
| Congo (Brazzaville) | 0.59 | 1.35 | 128.55 |
| Comoros | 0.07 | 0.26 | 262.9 |
| Cape Verde | 0.41 | 1.77 | 331.59 |
| Djibouti | 0.2 | 4.43 | 2083.46 |
| Eritrea | 0.19 | 0.21 | 8.48 |
| Ethiopia | 0.06 | 1 | 1515.05 |
| Gabon | 0.37 | 1.97 | 437.57 |
| Ghana | 0.21 | 2.14 | 918.89 |
| Guinea | 0.02 | 0.4 | 1926.91 |
| The Gambia | 0.05 | 0.71 | 1264.97 |
| Guinea-Bissau | 0.11 | 1.06 | 884.49 |
| Equatorial Guinea | 0.44 | 6.03 | 1259.03 |
| Kenya | 1.99 | 9.25 | 363.97 |
| Liberia | 0.05 | 1.09 | 2231.36 |
| Lesotho | 0.89 | 12.96 | 1361.87 |
| Madagascar | 0.02 | 0.03 | 63.04 |
| Mali | 0.19 | 0.24 | 27.1 |
| Mozambique | 0.05 | 4.75 | 9675.17 |
| Mauritania | 0.13 | 0.56 | 336.01 |
| Malawi | 0.31 | 3.5 | 1030.38 |
| Namibia | 14.51 | 48.34 | 233.28 |
| Niger | 0.01 | 0.1 | 601.27 |
| Nigeria | 0.07 | 1.16 | 1678.01 |
| Rwanda | 0.31 | 4.11 | 1217.52 |
| Senegal | 0.14 | 0.45 | 225.54 |
| Sierra Leone | 0.02 | 0.25 | 1167.02 |
| Somalia | 0.01 | 0.01 | -35.71 |
| South Sudan | 0.07 | 0.71 | 984.53 |
| São Tomé and Príncipe | 0.16 | 0.77 | 375.68 |
| eSwatini | 6.26 | 27.76 | 343.55 |
| Chad | 0.09 | 0.29 | 236.74 |
| Togo | 0.19 | 0.67 | 246.84 |
| Tanzania | 0.2 | 2.86 | 1324.96 |
| Uganda | 0.58 | 3.83 | 566.58 |
| South Africa | 7.94 | 25.71 | 223.83 |
| Zambia | 1.14 | 6.19 | 443.08 |
| Zimbabwe | 0.74 | 6.22 | 735.95 |

| **Table IIIf:** HIV Incidence Rates by country | | | |
| --- | --- | --- | --- |
| Location | 2000 | 2015 | Percent Change |
| Angola | 1.2 | 1.38 | 15.09 |
| Burundi | 1.93 | 0.27 | -86.15 |
| Benin | 1.06 | 0.36 | -65.99 |
| Burkina Faso | 0.96 | 0.2 | -79.22 |
| Botswana | 15.58 | 5.35 | -65.65 |
| Central African Republic | 4.17 | 1.71 | -58.96 |
| Côte d'Ivoire | 3.78 | 0.93 | -75.43 |
| Cameroon | 4.32 | 1.48 | -65.7 |
| DR Congo | 1.1 | 0.27 | -75.08 |
| Congo (Brazzaville) | 2.33 | 1.45 | -38.02 |
| Cape Verde | 0.6 | 0.3 | -50.48 |
| Djibouti | 2.71 | 0.88 | -67.42 |
| Eritrea | 1.07 | 0.23 | -78.8 |
| Ethiopia | 5.41 | 1.16 | -78.64 |
| Gabon | 3.3 | 1.79 | -45.6 |
| Ghana | 1.87 | 0.69 | -62.99 |
| Guinea | 1.19 | 0.88 | -25.72 |
| The Gambia | 1.73 | 1.09 | -37.31 |
| Guinea-Bissau | 2.38 | 1.01 | -57.34 |
| Equatorial Guinea | 3.51 | 5.28 | 50.57 |
| Kenya | 58.31 | 9.82 | -83.15 |
| Liberia | 1.41 | 0.65 | -53.95 |
| Lesotho | 18.98 | 11.67 | -38.52 |
| Madagascar | 0.17 | 0.2 | 14.97 |
| Mali | 1.04 | 0.42 | -59.56 |
| Mozambique | 7.9 | 7.77 | -1.61 |
| Malawi | 8.74 | 2.8 | -68.03 |
| Namibia | 11.67 | 4.03 | -65.44 |
| Niger | 0.5 | 0.12 | -75.26 |
| Nigeria | 6.29 | 3.22 | -48.72 |
| Rwanda | 2.65 | 0.62 | -76.66 |
| Senegal | 0.62 | 0.21 | -66.38 |
| Sierra Leone | 1.16 | 0.96 | -16.71 |
| Somalia | 0.7 | 0.19 | -72.45 |
| South Sudan | 1.4 | 1.06 | -24.31 |
| eSwatini | 23.42 | 8.78 | -62.52 |
| Chad | 1.35 | 0.71 | -47.23 |
| Togo | 3.27 | 0.78 | -76.16 |
| Tanzania | 4.53 | 1.45 | -67.98 |
| Uganda | 4.92 | 2.08 | -57.66 |
| South Africa | 23.45 | 9.8 | -58.2 |
| Zambia | 9.07 | 3.65 | -59.69 |
| Zimbabwe | 12.69 | 3.4 | -73.17 |

| **Table IIIg:** HIV Knowledge Scores by country | | | |
| --- | --- | --- | --- |
| Country | 2000 | 2015 | Percent Change |
| Angola | 0.41 | 0.67 | 65.27 |
| Burundi | 0.7 | 0.86 | 22.49 |
| Benin | 0.52 | 0.69 | 33.7 |
| Burkina Faso | 0.46 | 0.83 | 78.32 |
| Botswana | 0.69 | 0.81 | 17.99 |
| Central African Republic | 0.44 | 0.77 | 74.85 |
| Côte d'Ivoire | 0.57 | 0.72 | 26.97 |
| Cameroon | 0.53 | 0.76 | 43.05 |
| DR Congo | 0.57 | 0.66 | 15.43 |
| Congo (Brazzaville) | 0.55 | 0.77 | 39.34 |
| Comoros | 0.47 | 0.66 | 40.31 |
| Cape Verde | 0.62 | 0.82 | 32.4 |
| Djibouti | 0.57 | 0.75 | 31.83 |
| Eritrea | 0.66 | 0.82 | 23.66 |
| Ethiopia | 0.55 | 0.72 | 32.05 |
| Gabon | 0.63 | 0.82 | 28.78 |
| Ghana | 0.67 | 0.74 | 10.51 |
| Guinea | 0.53 | 0.66 | 25.12 |
| The Gambia | 0.5 | 0.76 | 52.74 |
| Guinea-Bissau | 0.33 | 0.76 | 133.15 |
| Equatorial Guinea | 0.5 | 0.74 | 49.07 |
| Kenya | 0.71 | 0.85 | 18.74 |
| Liberia | 0.48 | 0.71 | 47.87 |
| Lesotho | 0.64 | 0.82 | 29 |
| Madagascar | 0.43 | 0.73 | 69.04 |
| Mali | 0.43 | 0.63 | 47.98 |
| Mozambique | 0.5 | 0.73 | 46.45 |
| 0Mauritania | 0.32 | 0.51 | 56.55 |
| Malawi | 0.71 | 0.85 | 20.75 |
| Namibia | 0.73 | 0.85 | 16.65 |
| Niger | 0.39 | 0.65 | 66.03 |
| Nigeria | 0.49 | 0.67 | 36.35 |
| Rwanda | 0.7 | 0.89 | 27.05 |
| Senegal | 0.59 | 0.69 | 16.34 |
| Sierra Leone | 0.39 | 0.67 | 72.86 |
| Somalia | 0.2 | 0.76 | 272.93 |
| South Sudan | 0.27 | 0.43 | 57.14 |
| São Tomé and Príncipe | 0.55 | 0.85 | 55.19 |
| eSwatini | 0.72 | 0.91 | 26.83 |
| Chad | 0.43 | 0.52 | 20.91 |
| Togo | 0.61 | 0.75 | 22.89 |
| Tanzania | 0.67 | 0.84 | 24.57 |
| Uganda | 0.66 | 0.85 | 28.72 |
| South Africa | 0.66 | 0.76 | 15.18 |
| Zambia | 0.68 | 0.84 | 23.73 |
| Zimbabwe | 0.73 | 0.88 | 21.42 |

| **Table IIIh:** Lag Distributed Income by country | | | |
| --- | --- | --- | --- |
| Country | 2000 | 2015 | Percent Change |
| Angola | 2842.03 | 6337.53 | 122.99 |
| Burundi | 713.03 | 731.79 | 2.63 |
| Benin | 1441.75 | 1818.63 | 26.14 |
| Burkina Faso | 967.2 | 1443.75 | 49.27 |
| Botswana | 8945.1 | 14139.95 | 58.07 |
| Central African Republic | 886.47 | 734.87 | -17.1 |
| Côte d'Ivoire | 2780.36 | 2839.23 | 2.12 |
| Cameroon | 2277.41 | 2648.4 | 16.29 |
| DR Congo | 645.24 | 718.44 | 11.34 |
| Congo (Brazzaville) | 3696.95 | 5318.09 | 43.85 |
| Comoros | 1524.05 | 1417.79 | -6.97 |
| Cape Verde | 2962.24 | 5846.42 | 97.36 |
| Djibouti | 2449.89 | 2778.35 | 13.41 |
| Eritrea | 1286.8 | 1095.15 | -14.89 |
| Ethiopia | 587.11 | 1229.24 | 109.37 |
| Gabon | 15623.47 | 15466.49 | -1 |
| Ghana | 2120.62 | 3432.71 | 61.87 |
| Guinea | 1398.24 | 1356.17 | -3.01 |
| The Gambia | 1526.96 | 1541.7 | 0.97 |
| Guinea-Bissau | 1452.09 | 1370.14 | -5.64 |
| Equatorial Guinea | 4605.04 | 37505.77 | 714.45 |
| Kenya | 2165.58 | 2621.8 | 21.07 |
| Liberia | 457.44 | 753.55 | 64.73 |
| Lesotho | 1629.66 | 2560.44 | 57.12 |
| Madagascar | 1234.95 | 1245.48 | 0.85 |
| Mali | 1249.85 | 1670 | 33.62 |
| Mozambique | 501.14 | 993.95 | 98.34 |
| Mauritania | 2526.12 | 3403.48 | 34.73 |
| Malawi | 848.57 | 1000 | 17.85 |
| Namibia | 5780.49 | 9237.04 | 59.8 |
| Niger | 760.76 | 851.51 | 11.93 |
| Nigeria | 2041.22 | 5215.86 | 155.53 |
| Rwanda | 766.59 | 1460.57 | 90.53 |
| Senegal | 1786.8 | 2140.6 | 19.8 |
| Sierra Leone | 1081.01 | 1411.3 | 30.55 |
| Somalia | 458.11 | 479.56 | 4.68 |
| South Sudan | 2815.05 | 2959.04 | 5.11 |
| São Tomé and Príncipe | 1885.55 | 2721.91 | 44.36 |
| eSwatini | 6282.13 | 7955.62 | 26.64 |
| Chad | 1172.43 | 1997.65 | 70.39 |
| Togo | 1265.39 | 1263 | -0.19 |
| Tanzania | 1274.02 | 2195.67 | 72.34 |
| Uganda | 1050.51 | 1752.51 | 66.83 |
| South Africa | 8891.21 | 11920.16 | 34.07 |
| Zambia | 1719.67 | 3325.17 | 93.36 |
| Zimbabwe | 3047.29 | 1730.71 | -43.2 |

| **Table IIIi:** Non-partner sexual violence by country | | | |
| --- | --- | --- | --- |
| Country | 2000 | 2015 | Percent Change |
| Angola | 0.09 | 0.11 | 19.5 |
| Burundi | 0.13 | 0.14 | 5.5 |
| Benin | 0.05 | 0.05 | 9.38 |
| Burkina Faso | 0.04 | 0.05 | 5.37 |
| Botswana | 0.07 | 0.07 | -3.21 |
| Central African Republic | 0.09 | 0.09 | 5.85 |
| Côte d'Ivoire | 0.05 | 0.05 | -1.66 |
| Cameroon | 0.05 | 0.06 | 7.79 |
| DR Congo | 0.07 | 0.08 | 6.26 |
| Congo (Brazzaville) | 0.11 | 0.1 | -8.78 |
| Comoros | 0.07 | 0.07 | -4.79 |
| Cape Verde | 0.05 | 0.06 | 11.41 |
| Djibouti | 0.12 | 0.11 | -8.25 |
| Eritrea | 0.14 | 0.14 | 4.26 |
| Ethiopia | 0.12 | 0.14 | 14.5 |
| Gabon | 0.13 | 0.15 | 12.94 |
| Ghana | 0.09 | 0.1 | 13.2 |
| Guinea | 0.04 | 0.05 | 19.16 |
| The Gambia | 0.04 | 0.04 | 8.91 |
| Guinea-Bissau | 0.05 | 0.05 | 8.4 |
| Equatorial Guinea | 0.11 | 0.12 | 12.86 |
| Kenya | 0.13 | 0.12 | -8.62 |
| Liberia | 0.05 | 0.04 | -2.15 |
| Lesotho | 0.09 | 0.09 | -2.29 |
| Madagascar | 0.13 | 0.13 | 0.66 |
| Mali | 0.03 | 0.03 | 10.77 |
| Mozambique | 0.21 | 0.24 | 16.61 |
| Mauritania | 0.05 | 0.06 | 13.7 |
| Malawi | 0.16 | 0.18 | 10.99 |
| Namibia | 0.06 | 0.06 | 4.9 |
| Niger | 0.04 | 0.05 | 11.02 |
| Nigeria | 0.06 | 0.07 | 9.71 |
| Rwanda | 0.19 | 0.17 | -12.65 |
| Senegal | 0.05 | 0.05 | 2.12 |
| Sierra Leone | 0.05 | 0.06 | 20.03 |
| Somalia | 0.12 | 0.11 | -9.73 |
| South Sudan | 0.12 | 0.12 | -1.49 |
| São Tomé and Príncipe | 0.05 | 0.05 | -0.3 |
| eSwatini | 0.46 | 0.43 | -4.76 |
| Chad | 0.04 | 0.04 | 11.13 |
| Togo | 0.06 | 0.06 | -5.39 |
| Tanzania | 0.23 | 0.25 | 5.82 |
| Uganda | 0.19 | 0.21 | 12.84 |
| South Africa | 0.1 | 0.1 | 0.71 |
| Zambia | 0.13 | 0.12 | -9.43 |
| Zimbabwe | 0.09 | 0.07 | -18.31 |

| **Table IV** Sums of squares for each covariate and model | | | | | | | |
| --- | --- | --- | --- | --- | --- | --- | --- |
| Model | Log Lag-Distributed Income | HIV Curative Care Spending | Knowledge Score | Years of Education Completed | Contraception Prevalence | Non-partner Sexual Violence | ART Coverage |
| log_ldi | 0.51 |  |  |  |  |  |  |
| log_spend | 0.5 | 5.5 |  |  |  |  |  |
| kw_score | 0.63 | 5.49 | 5.75 |  |  |  |  |
| educ_year_pc | 0.69 | 5.62 | 5.33 | 5 |  |  |  |
| contracept_prev | 0.67 | 5.64 | 5.31 | 5.02 | 0.14 |  |  |
| non_partner_sv | 0.68 | 5.65 | 5.36 | 5.02 | 0.14 | 0.18 |  |
| art_rate | 0.99 | 5.83 | 5.97 | 5.13 | 0.15 | 0.23 | 1.25 |

| **Table V** Model fit statistics when removing covariates | | | |
| --- | --- | --- | --- |
| Model | AIC | BIC | Deviance |
| Lagged distributed income, logged HIV Curative Care Spending + Knowledge Score + Years of Education Completed | -342.82 | -302.01 | -360.82 |
| Years of Education Completed | -343 | -311 | -356.8 |
